# Definition of the traditional African diet: a scoping review

**DOI:** 10.3389/fnut.2025.1651945

**Published:** 2025-09-12

**Authors:** Jizhao Niu, Nina Frances Ockendon-Powell, Toluwanimi Ann Alonge, Angeliki Papadaki

**Affiliations:** ^1^School for Policy Studies, Faculty of Arts, Law and Social Sciences, University of Bristol, Bristol, United Kingdom; ^2^School of Biological Sciences, Faculty of Health and Life Sciences, University of Bristol, Bristol, United Kingdom

**Keywords:** African diet, traditional diets, definition, diet habit, scoping review, food pattern

## Abstract

**Background:**

Traditional diets are increasingly studied for their purported health and environmental benefits. Promoting the traditional African diet (TrAfDi) could be a promising means of addressing the impacts of rapid dietary transitions in African countries. However, there is no consistent definition of this traditional dietary pattern. The aim of this scoping review was therefore to systematically explore, for the first time, the definition of the TrAfDi, as reported in the literature to date.

**Methods:**

Seven databases were searched, up to January 16^th^, 2023, for peer-reviewed studies and gray literature describing the TrAfDi. One reviewer screened articles, extracted data, and assessed article quality; an independent reviewer screened 10% of titles, abstracts, and full-text articles. Reporting followed the Preferred Reporting Items for Systematic reviews and Meta-Analyses for Scoping Reviews (PRISMA-ScR) guidelines.

**Results:**

We included 45 studies that defined a TrAfDi. The food groups characterizing the TrAfDi include cereals and their products, pulses, seeds and nuts and their products, and vegetables and their products. Other groups, cited less frequently, include fruits and their products, and roots, tubers, plantains, and their products. Maize was the most highly cited food item. Other, lesser-cited food items include cassava, cowpeas, fish, fruit, legumes, millet, and sorghum. Minor regional differences in the TrAfDi were observed when studies were segregated according to United Nations classifications. Differences were mainly observed between Western Africa and all other African regions, which, between them, do not appear to exhibit significant variation in the most frequently cited food groups. Few studies reported the quantities of foods consumed and the frequency of consumption.

**Conclusion:**

These findings provide important initial evidence on what may constitute a TrAfDi and indicate features of its regional characteristics, and are relevant to the development of public health policies seeking to tackle challenges of food insecurity, obesity, and non-communicable diseases in Africa. These will underpin future research to assess the TrAfDi's health and environmental impact, and to understand the cultural implications of shifting dietary patterns resulting from climatic, economic, and other factors. Future studies should also aim to strengthen regional representativeness and establish the quantities of foods that characterize this dietary pattern.

**Systematic review registration:**

https://osf.io/kvu2n.

## 1 Introduction

Traditional diets have received immense interest in past decades due to their potential beneficial effects on health and environmental sustainability (1, 2). Defined as the dietary patterns “*of a particular culture that have been consumed over generations, are aligned with the cultural and/or religious preferences and environmental availability of the region, and prioritize home-cooked meals featuring biodiverse foods that were commonplace before the advent of highly processed foods and industrial monoculture agriculture”* (3), traditional diets are characterized by high consumption of plant foods and low consumption of animal products, processed foods, sugar, and salt, and highlight the consumption of seasonal and regional products (4, 5). Several systematic reviews have suggested beneficial health outcomes of consuming traditional diets, such as the Mediterranean (6, 7), Chinese (8), Nordic (6, 9, 10), Japanese, and Atlantic (6) diets, while adherence to the traditional Mexican diet has also been suggested to hold promise in improving several non-communicable disease-related outcomes (11, 12). Because traditional diets are biodiverse and culturally acceptable, and promote economic and environmental security by optimizing human and natural resources, they can also be considered sustainable (1, 13–17). Promoting traditional diets is therefore of the utmost importance.

The African continent, with its diverse ethnic groups, cultures, traditions, geography, and climate, has seen an increase in the prevalence of non-communicable diseases (NCDs), which are responsible for 37% of deaths (18). Approximately 19 million people in Africa live with diabetes (18), whereas over a million deaths annually are attributed to cardiovascular diseases in sub-Saharan countries (19). Despite under-nutrition and underweight remaining highly prevalent, obesity rates are high in many countries of the continent (20, 21). It is expected that the burden from NCD morbidity and mortality will exceed that of infectious diseases by 2035 (19, 21).

This rise in NCDs has been linked to shifts in dietary patterns driven by urbanization, globalization, rapid economic development, and technological advancement, which have collectively contributed to the adoption of less healthy diets (19, 22–25). Traditional diets often fail to resist urbanization and globalization due to a combination of increased availability and aggressive marketing of processed foods, changing work and lifestyle patterns that reduce time for home-cooked meals, and the perception that Western diets reflect social and economic progress. Rapid urbanization often leads to significant changes in the food environment, including increased access to ultra-processed foods, the proliferation of supermarkets and fast-food outlets, and shifts in cultural norms and preferences (26). Globalization introduces new dietary practices and aggressive food marketing strategies that further shape consumer choices (27). At the same time, approximately 35% of the population in Africa lives in extreme poverty (28), and the continent is among the most vulnerable to climatic and environmental fluctuations that directly impact food cultivation, production, and availability (29, 30). These factors contribute to limited dietary diversity and food insecurity, as well as a nutritional transition that moves populations away from traditional diets (31). Although the EAT-Lancet diet has been promoted in Africa as a healthy and sustainable model, recent research reported extremely low adherence with this diet, highlighting the need to promote more culturally acceptable and practical dietary patterns in the region (32). In this context, exploring the traditional African diet (TrAfDi) is essential, not only to promote efforts for reducing the burden of NCDs, but also to identify culturally and nutritionally important foods that may be more resilient to environmental and food system fluctuations. This aligns with a growing body of scholarship emphasizing the potential of traditional diets to contribute to sustainable and healthy eating patterns both within Africa and globally (33, 34).

A recent narrative perspective study explored the African heritage diet by describing food patterns across the African diaspora, including those consumed by people residing in the African continent, the American South, the Caribbean, and South America (3). This study mainly focused on the traditional foods brought by African descendants to the New World, alongside those they adopted, instead of concentrating on the culinary traditions characterizing the continental African diet. This review approaches the TrAfDi as a regional, rather than country-specific, dietary model, similar to how the Mediterranean diet has been conceptualized in the literature (3). Similar to the Mediterranean diet, however, which encompasses diverse but related food cultures across multiple countries (3), the TrAfDi likely reflects broad patterns that may transcend national borders while still retaining local relevance. Within the scope of this review, **traditional diets** refer broadly to long-term established dietary patterns embedded within African cultures (3), whereas **indigenous diets** are defined more specifically as the traditional food systems of indigenous populations, typically rooted in local ecological knowledge, regionally available ingredients, and ancestral practices (35). In this review, **indigenous diets are considered a subset of traditional diets**, as they represent enduring, culturally grounded foodways that contribute to a more comprehensive understanding of the TrAfDi across different regions.

To the best of our knowledge, no study to date has reviewed the evidence base documenting what constitutes the TrAfDi. Therefore, the aim of this scoping review was to explore how the TrAfDi is defined in the literature to date. A specific objective was to document the food groups and food items that characterize the TrAfDi, as well as their frequency of consumption, and/or amounts consumed as part of this traditional dietary pattern. Given the significant changes in food culture and dietary practices caused by colonial influences, including the decline of indigenous crops (relating to particular locations) and the rise of imported foods, which contribute to the nutrition transition (36, 37), this review also explored how the TrAfDi has evolved over time. Specifically, we examined descriptions in the literature of dietary patterns before, during, and after colonization to better understand how historical processes might have shaped contemporary food systems and nutritional landscapes across the continent.

## 2 Methods

### 2.1 Study design

Scoping reviews can be effective in mapping key concepts and identifying research gaps based on the existing literature (38). Additionally, this type of review can detail the extent of literature on a topic, particularly when the subject matter is complex, involves diverse study methodologies, or is being reviewed for the first time (39). Considering the potential breadth of the evidence base exploring the definition of the TrAfDi, a scoping review was deemed the most appropriate method to address the aims and objectives of the study. We adopted established methodological frameworks and reported the review following the Preferred Reporting Items for Systematic Reviews and Meta-Analyses for Scoping Reviews (PRISMA-ScR) guidelines (40) (Supplementary material I, Supplementary Table S1). The review protocol was registered in the Open Science Framework platform (https://osf.io/kvu2n).

### 2.2 Identifying relevant studies

The search strategy was developed by author JN, in consultation with authors NOP and AP, as well as the University's subject librarian. The following databases were searched from inception until 16 January 2023: African Journal Archive, SciELO Citation Index on Web of Science, International Bibliography of the Social Sciences (IBSS), Web of Science Core Collection, PubMed, and Cochrane Library. Searches were limited to articles published in English and involving humans, and used a combination of the following keywords and Boolean operators: Africa^*^ [AND] tradition^*^ OR region^*^ OR nation^*^ OR native OR indigenous [AND] diet^*^ OR “diet^*^ pattern^*^” OR “food pattern^*^” OR “eating pattern^*^” OR “food habit^*^” OR “eating habit^*^” OR “diet habit^*^” OR cuisine. During the search strategy development, the term “indigenous” was observed to be used frequently in the literature to describe locally rooted and culturally specific foods, particularly in traditional dietary practices. This informed the inclusion of this term within the search strategy. Additionally, the reference lists of eligible papers were checked to identify further potentially eligible reports. Gray literature, such as governmental reports or policy documents, was also searched via the OVERTON database. The detailed search strategy employed for each database can be found in Supplementary material I, Supplementary Table S2.

### 2.3 Study selection

Papers, as well as gray literature, were eligible for inclusion if they described the TrAfDi and reported on observational studies (qualitative or quantitative), experimental studies, and reviews (whether systematic or not). We excluded study protocols, conference abstracts, news, and editors' comments or editorials. As the aim of this scoping review was to explore how the TrAfDi is defined in the literature, papers that identified dietary patterns of African populations but did not specifically recognize them as “traditional African,” or characteristic of the continent or its countries, were excluded (e.g., those identifying “prudent” or “healthy” dietary patterns). Additionally, only papers that described a whole TrAfDi were included; papers describing or analyzing single traditional African foods were ineligible. Papers were also eligible if they referred to adults identified as African or those of African ancestry. Non-English articles, those for which the full text could not be retrieved, and those containing insufficient information for data extraction, were also excluded.

The screening of identified papers was conducted in Rayyan (41). After eliminating duplicates, the titles and abstracts of all remaining papers were assessed for relevance by the author JN, and a randomly selected subset of these (10%) was also independently screened by the author TAA, to promote consistency in applying the inclusion criteria. For full-text screening, both JN and TAA independently screened all full texts to determine final eligibility. Discrepancies at any stage of the screening process were resolved through discussion and adjudication with authors NOP and AP, until consensus was reached.

### 2.4 Charting the data and quality assessment

Data extraction was conducted independently by authors JN and TAA, using a modified template from a recent systematic review that explored the definition of the traditional Mexican diet (12). This modified template was verified for relevance by the authors and utilized to extract essential information from the included reports, such as methodological details and descriptions of the TrAfDi, including its food groups and items, and their quantities, when available (Supplementary material I, Supplementary Table S3). The corresponding authors of included reports were contacted to obtain any information deemed important for the data extraction process, which could not be found in the original sources.

The quality of included reports was assessed by author JN, using an adapted version of a quality assessment tool from earlier systematic reviews that explored traditional dietary patterns (12, 42). This evaluated the transparency, relevance, and representativeness of the deviation of the TrAfrDi in the literature, and included items such as the reporting of food items/food groups included in the diet, the proportions, quantities, or frequencies of foods included in the TrAfrDi, and the reporting of the geographical area(s) that the TrAfrDi represented (Supplementary material I, Supplementary Table S4).

### 2.5 Collating, summarizing, and reporting the results

Raw data from all included studies were extracted into an Excel spreadsheet. Similar to a recent systematic review exploring the definition of the traditional Chinese diet (8), food item terms were manually curated into standardized forms to account for variations in the reporting of related foods. For example, “fermented maize products” was reported as “Maize products, fermented;” “whole mealies” and “stamp mealies” were reported as “Mealies, whole” and “Mealies, stamp,” respectively. All reported food items per study (Supplementary material II, Supplementary Table S5), and assignment of cited food items to food groups per study (Supplementary material II, Supplementary Table S6) were then compiled using custom Python scripts. The categorization of food groups and assignment of individual food items into food groups (Supplementary material I, Supplementary Tables S7, S8) were based on the Food and Agriculture Organization/World Health Organization Global Individual Food Consumption Data Tool. A further food group was added to this set, “indigenous dishes or beverages,” to accommodate the traditional foods reported by various studies that were specific to certain indigenous communities or regions. These items typically comprised mixtures of food ingredients or interchangeable main food items. Where studies cited certain foods that could be considered food groups, these were assigned to the relevant food group, as per the food items. The frequencies of food items and food group citations in the included reports were then calculated to summarize the definition of the TrAfrDi. Quartiles provide a concise summary of data distributions and are widely recognized as a standardized method for presenting results (43). Therefore, the food citation analysis in this study used quartiles as thresholds to describe citation frequency patterns: food groups with citation frequencies of at least 25%, 50%, and 75% across the included reports were documented (8). Food groups with citation frequencies over 75% were considered characteristic of the TrAfDi. However, lower thresholds of 10%, 25%, and 50% were used for food items, as these citation frequencies were typically lower, due to distribution across a wide variety of food item terms. Amounts of food groups/food items were captured, where available, and are reported in Supplementary material I, Supplementary Table S9.

The African continent is divided into Northern Africa, Southern Africa, Central Africa, Eastern Africa, and Western Africa (44). Regional dietary differences were accounted for by reporting food groups from different regions separately. For studies that specified a region, subgroup analyses were conducted, which included the calculation of food group citations for these five African regions. Where studies specified more than one region (for example, Western Africa and Southern Africa), food groups were included in the calculations for both regions. Studies that did not specify regions (*n* = 2) were considered to represent the entirety of the African continent.

## 3 Results

**Figure 1:**
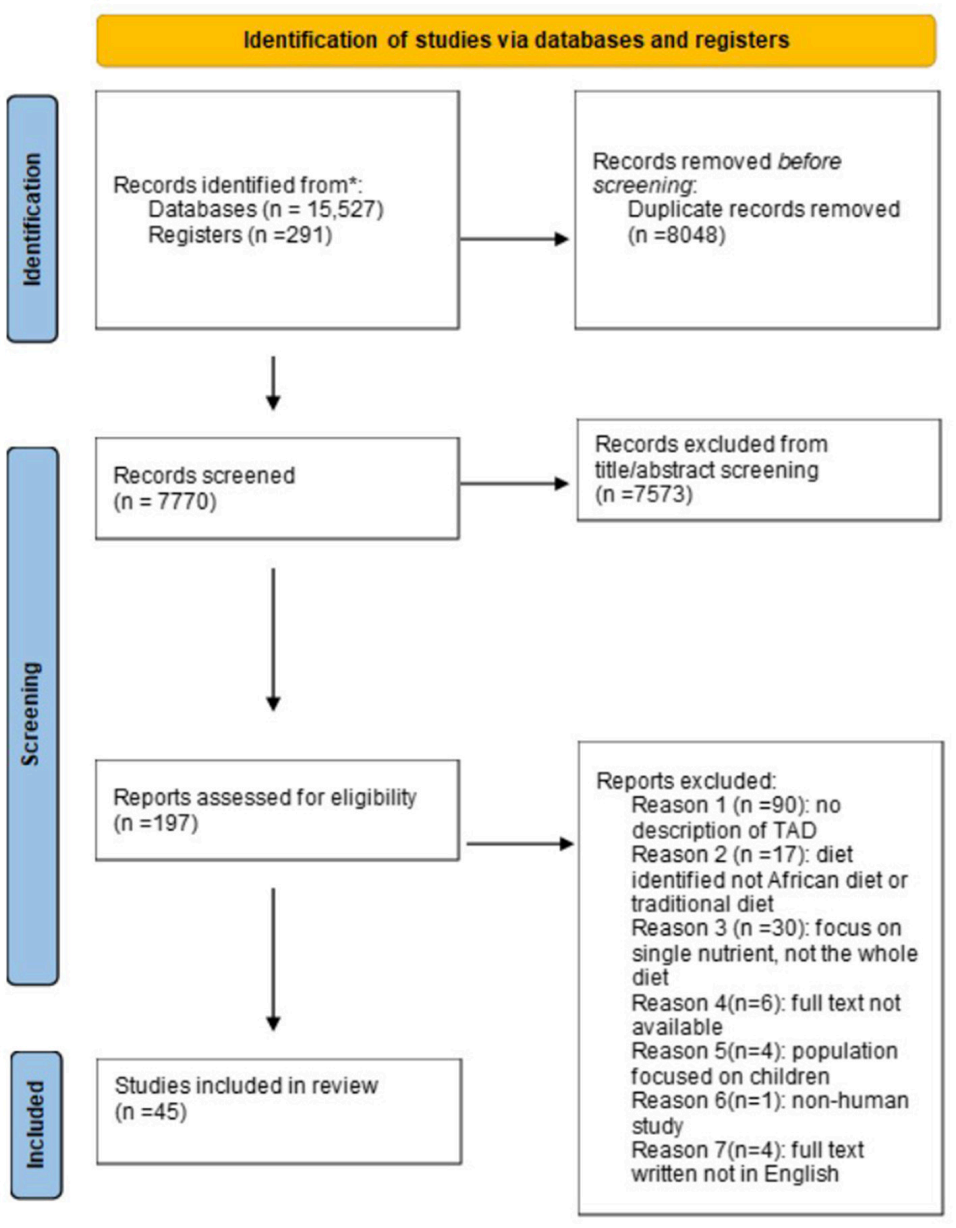
Flowchart of the search and selection process.

A total of 15,818 reports were identified, and 7,770 remained for title and abstract screening after removal of duplicates. A further 7,573 reports were excluded because they were not relevant to this review's aim. The full text of 197 reports was then read, and 152 were excluded, mostly because they did not provide a definition of the TrAfDi. As a result, 45 reports were used for analysis (Figure 1).

### 3.1 Study characteristics

The characteristics of the included studies are presented in Supplementary material I, Supplementary Table S10. Of the 45 studies, 16 were literature reviews (45–60), 10 were cross-sectional studies (61–70), 7 were qualitative studies (including focus groups and interviews, field work, and semi-structured interviews), 3 were case–control studies (71–73), 2 were mixed-methods (including transdisciplinary) (74, 75), 2 were cohort studies (76, 77), and 2 were observational studies (78, 79). The remainder included one narrative review (84), one systematic review (87), and one study report (80).

Of the 26 empirical research studies, the majority (*n* = 14) utilized quantitative methods to define the TrAfDi, by using principal component analysis (PCA) or cluster analysis (61–64, 66–68, 70–73, 76, 77, 79). These included eight cross-sectional studies (61–64, 66–68, 70), one observational study (79), two cohort studies (76, 77), and three case–control studies (71–73). Additionally, qualitative methods were applied in 12 studies to derive the TrAfDi (45, 47, 51, 53, 55, 58, 75, 81–85). These methods involved providing participants with a list of food items and food groups for selection, and discussion to identify the most representative or accepted food items and groups, as well as using previous literature as a reference to define the TrAfDi. All studies referring to the whole African continent (*n* = 5) were narrative studies. Of the studies referring to Eastern Africa (*n* = 10), 30% were narrative syntheses, 10% were qualitative studies, and 60% were quantitative studies. Of the studies referring to Western Africa (*n* = 12), 25% were narrative syntheses, and 75% were quantitative studies. Of studies referring to Southern Africa (*n* = 21), 43% were narrative syntheses, 24% were qualitative studies, 24% were quantitative studies, and 10% were mixed-method studies. The single study referring to Northern Africa was a narrative synthesis.

Most of the studies (*n* = 30; 66.7%) did not report the years/period represented by the data. Ten studies represented data from 2009 to 2019 (61, 62, 64, 70–73, 75, 76, 81). Four studies represented data from the early to the late 1900s (50, 51, 65, 66). One study reported data from 5,000 years ago, 1,000 years ago, and the colonial period (55).

### 3.2 Definition of the overall TrAfDi

The most frequently cited food item, cited by >50% of studies, was maize, while cassava (manioc), cowpeas (*Vigna unguiculata*), fish, fruit, legumes, millet, sorghum, and vegetables were cited by >25% of studies (Table 1a and Supplementary material II, Supplementary Table S5). When all cited food items were assigned to food groups, the food groups that characterize the TrAfDi (reported by >75% of studies) were cereals and their products, pulses, seeds and nuts and their products, and vegetables and their products. Food groups cited by >25% and >50% of studies are listed in Table 1b and Supplementary material II, Supplementary Table S6.

**Table 1:** Most-cited food items and food groups reported in the included studies.

**a. Food items cited by at least 10%, 25%, and 50% of the studies (*****n*** = 45)^*^		
>**10%**	>**25%**	>**50%**
Amaranth; Bambara groundnuts (*Vigna sub-terranea*); Bananas; Baobab (*Adansonia digitata*); Beans; Beef; Bread, white or dark; Cereal grains; Chicken; Grains; Green leafy vegetables; Groundnuts (arachis hypogaea); Mangoes; Meat; Milk; Nuts; Offal; Okra; Plantain; Potatoes; Pulses; Pumpkin; Rice; Roots; Seeds; Spinach; Sweet potato; Tomatoes; Tubers; Watermelon; Yams (dioscorea)	Cassava (manioc); Cowpeas (*Vigna unguiculata*); Fish; Fruit; Legumes; Millet; Sorghum; Vegetables	Maize
**b. Food groups cited by at least 25%, 50%, and 75% of the studies (*****n*** = **45)**		
>**25%**	>**50%**	>**75%**
- Fats and oils (26.7%) - Fish, shellfish, and their products (26.7%) - Meat and meat products (46.7%) - Milk and milk products (31.1%)	- Fruits and their products (66.7%) - Roots, tubers, plantains, and their products (66.7%)	- Cereals and their products (80.0%) - Pulses, seeds, and nuts and their products (75.6%) - Vegetables and their products (80.0%)

^*^Detailed description of these foods can be found in Supplementary material I, Supplementary Table S8.

Considering the relative citation frequency of the different food groups, some groups were reported notably different by certain study designs/ types (see Supplementary material II): “Beverages” were reported more frequently by narrative syntheses; “Cereals and their products” were reported less frequently by qualitative studies; “Fats and oils” and “fish, shellfish and their products” were reported more frequently by narrative syntheses and quantitative studies; “Fruits and their products” were reported more frequently by mixed method studies; “Indigenous dishes or beverages” were reported more frequently by qualitative and mixed method studies; “Insects, grubs and their products” were reported more frequently by qualitative studies; “Meat and meat products” were reported less frequently by qualitative studies; “Milk and milk products” were reported more frequently by narrative syntheses and quantitative studies; “Pulses, seeds and nuts and their products” were reported less frequently by quantitative studies; “Roots, tubers, plantains and their products” were reported less frequently by qualitative studies; and “Vegetables and their products” were reported less frequently by quantitative studies.

In total, 10 studies reported indigenous foods and beverages. In this context, the term “indigenous” refers to dishes/beverages which are characteristic of a specific country or region, named using local languages, and which combine multiple food items. These included 24 items, which were collated into a separate food group (“indigenous dishes or beverages”), supplementary to the FAO-provided food groups (Supplementary material II, Supplementary Table S6, Supplementary material I, Supplementary Tables S7, S8). A total of 38% of these were identified by studies considering the whole of Africa, 42% were identified by studies of Southern Africa, and the remaining 21% were identified from studies of Western Africa. For instance, kenkey, made from maize, has been regarded as a typical indigenous staple dish in Ghana (79). Additionally, burukutu, an alcoholic beverage brewed from Guinean corn and millet, has been popular in African regions and is made using traditional methods (45). These ‘indigenous dishes or beverages' are commonly prepared using traditional methods and have significant cultural meaning in African societies. As such, in this review, they are considered a subset of the TrAfDi.

### 3.3 Definition of the TrAfDi according to different geographical regions

**Figure 2:**
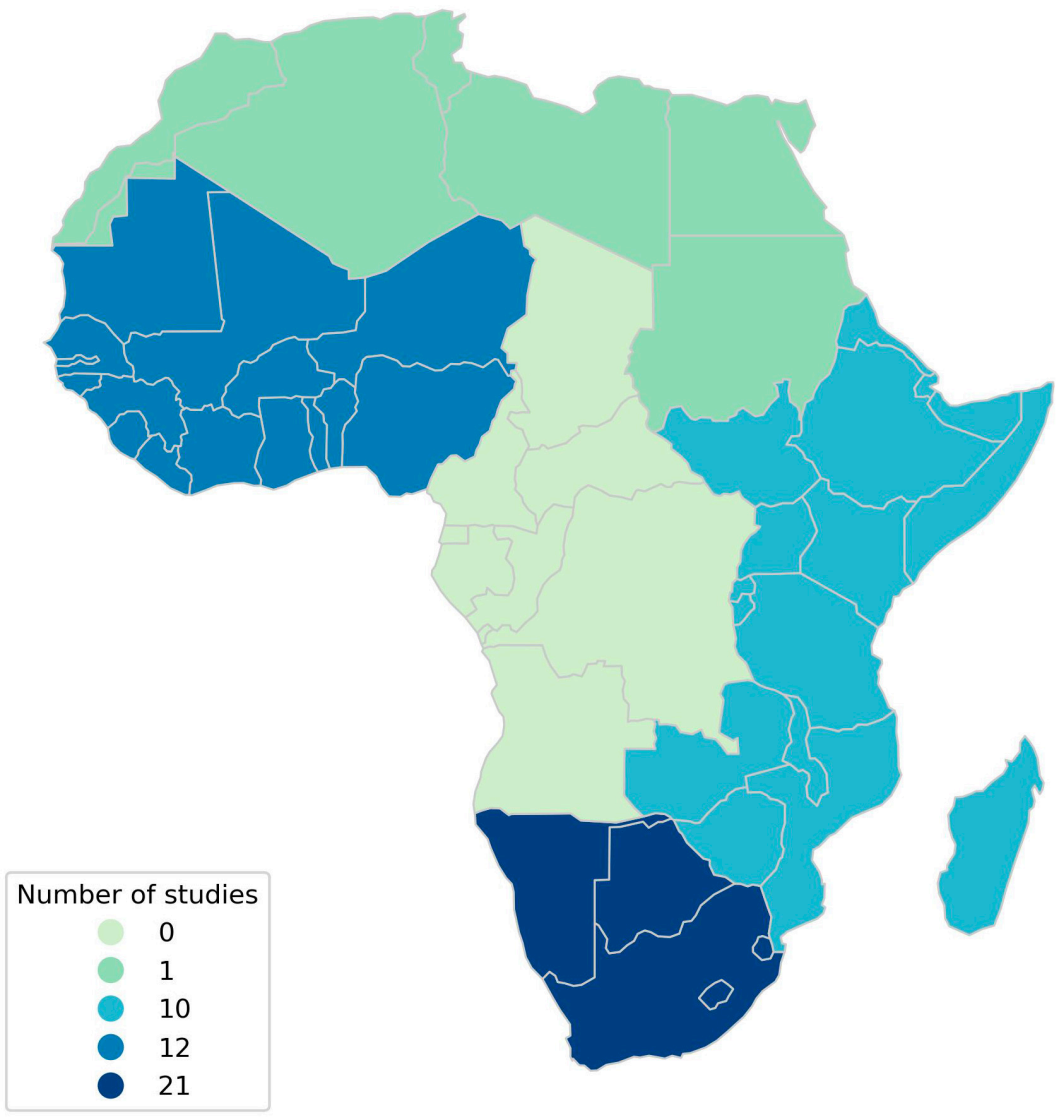
Map illustrating the regional distribution of studies across the continent of Africa. The five studies that referred to all of Africa without specifying a region were excluded from the regional totals used to create the map.

The majority of reviewed studies (*n* = 43) reported foods from particular regions of Africa: 5 studies referred to all of Africa, 1 referred to Northern Africa, 10 to Eastern Africa, 21 to Southern Africa, and 12 to Western Africa (Figure 2; the five studies that referred to all of Africa without specifying a region are not shown in Figure 2). As four studies referred to two regions of Africa (50, 53, 71, 78), the food groups reported by these studies were assigned to both regions referenced (and therefore the total number of studies referring to regions appears higher than the total number of studies included). The studies referring to regions of Africa reported similar food groups to the overall TrAfDi definition (Table 2). Cereals and their products were reported by at least 75% studies referencing each African region. In contrast to the definition of the overall TrAfDi, roots, tubers, plantains, and their products were reported by at least 75% of studies reporting on regional diets (50% for Eastern Africa).

**Table 2:** Most-cited food groups reported in the included studies, by African region.

**a. Food groups listed in at least 25%, 50%, and 75% of the studies referring to all of Africa (*****n*** = **5)**		
>**25%**	>**50%**	>**75%**
- Beverages - Fish, shellfish, and their products - Indigenous dishes or beverages	- Milk and milk products - Pulses, seeds, and nuts and their products - Spices and condiments	- Cereals and their products - Fruits and their products - Meat and meat products - Roots, tubers, plantains, and their products - Vegetables and their products
**b. Food groups listed in the study referring to Northern Africa (*****n*** = **1)**		
Beverages; Cereals and their products; Eggs and their products; Fats and oils; Fish, shellfish and their products; Fruits and their products; Meat and meat products; Milk and milk products; Pulses, seeds and nuts and their products; Vegetables and their products.		
**c. Food groups listed in at least 25%, 50%, and 75% of the studies referring to Eastern Africa (*****n*** = **10)**		
>**25%**	>**50%**	>**75%**
- Meat and meat products - Milk and milk products	- Cereals and their products - Fruits and their products - Pulses, seeds, and nuts and their products - Roots, tubers, plantains, and their products - Vegetables and their products	
**d. Food groups listed in at least 25%, 50%, and 75% of the studies referring to Southern Africa (*****n*** = **21)**		
>**25%**	>**50%**	>**75%**
- Indigenous dishes or beverages - Meat and meat products	- Fruits and their products - Roots, tubers, plantains, and their products	- Cereals and their products - Pulses, seeds, and nuts and their products - Vegetables and their products
**e. Food groups listed in at least 25%, 50%, and 75% of the studies referring to Western Africa (*****n*** = **12)**		
>**25%**	>**50%**	>**75%**
- Eggs and their products - Milk and milk products	- Fats and oils - Fish, shellfish, and their products - Fruits and their products - Meat and meat products - Pulses, seeds, and nuts and their products - Vegetables and their products	- Cereals and their products - Roots, tubers, plantains, and their products

At least 75% of studies referring to all of Africa (*n* = 5) reported cereals and their products, fruits and their products, meat and meat products, roots, tubers, plantains and their products, and vegetables and their products to be characteristic of the TrAfDi (45, 47, 57, 84, 86). The only study to refer to Northern Africa (*n* = 1) reported beverages, cereals and their products, eggs and their products, fats and oils, fish, shellfish and their products, fruits and their products, meat and meat products, milk and milk products, pulses, seeds and nuts and their products, and vegetables and their products to be characteristic of the diet of this region (87). None of the reviewed studies referred specifically to Central Africa. For studies referring to Eastern Africa (*n* = 10), no food groups were reported in at least 75% of the studies (53, 55, 60, 61, 65, 66, 68, 69, 71, 88). Food groups reported by at least 50% of studies for Eastern Africa comprised cereals and their products, fruits and their products, pulses, seeds and nuts and their products, roots, tubers, plantains and their products, and vegetables and their products. At least 75% of studies referring to Southern Africa (*n* = 21) reported cereals and their products, pulses, seeds and nuts and their products, and vegetables and their products (48–53, 56, 58, 59, 73–78, 80–83, 85, 89). At least 75% of studies referring to Western Africa (*n* = 12) reported cereals and their products, and roots, tubers, plantains and their products, to be characteristic of the Western region (46, 50, 54, 62–64, 67, 70–72, 78, 79).

### 3.4 Definition of the TrAfDi according to different time periods

As previously mentioned, only 15 studies specified the period/time their dietary data represented. Of these, 14 primarily focused on food groups/items consumed during and/or after colonization (50, 51, 61, 62, 64–66, 70–73, 75, 76, 81). The findings from these 14 studies were consistent with the overall results for the TrAfDi, highlighting that the most frequently reported food groups included cereals and their products, pulses, seeds and nuts and their products, and vegetables and their products (Table 3).

**Table 3:** Most-cited food groups reported in the included studies, by time period.

**a. Food groups listed in at least 25%, 50% and 75% of the studies referring to during and/or after colonization (*****n*** = **14)**		
>**25%**	>**50%**	>**75%**
- Oils - Fish, shellfish, and their products - Indigenous dishes or beverages - Milk and milk products	- Meat and meat products - Roots, tubers, plantains, and their products	- Cereals and their products - Fruits and their products - Pulses, seeds, and nuts and their products - Vegetables and their products
**b. Food groups listed in the study referring to before colonization (*****n*** = **1)**		
5,000 years ago: wild cereals; wild eggs; wild fish; wild plant foods; wild insects. 1,000 years ago: meat and meat products; cereals and millet; beans; spices and condiments; vegetables and their products; pulses, seeds, and nuts and their products.		

Only one study examined foods consumed before the colonial period (55). It reported that around 5,000 years ago, the most commonly consumed food sources were wild products, such as wild game, birds and eggs, fish, insects (e.g., grasshoppers and termites), and wild plant foods (e.g., fruits, nuts, and honey). Approximately 1,000 years ago, during the era of African agriculturalism, the traditional diet had evolved to include products from pastoral animals (e.g., milk, offal, and blood), beans, sorghum, pearl millet, and other starchy foods. This study also indicated that since the colonial period, imported crops, such as maize, rice, banana, tomato, and pumpkin, have increasingly influenced and shaped the TrAfDi.

### 3.5 Quantities of food consumption and cooking methods in the TrAfDi

Only four studies reported food consumption quantities, and all of them focused on food group consumption. One study reported the percentage of total energy intake contributed by different food groups (79), two reported average daily food group consumption (67, 80), and one reported food group consumption by quintiles of weekly servings (71). Three of these refer to Western Africa (with one also referring to Eastern Africa) (67, 71, 79), and one refers to Southern Africa (80). These studies utilized different methods for defining and assessing food groups, and the reported amounts varied significantly (Supplementary material I, Supplementary Table S9).

Five studies mentioned cooking methods. Three studies, referring to Southern Africa, reported boiling (*n* = 3), mashing (*n* = 2), fermenting (*n* = 2), and steaming (*n* = 1) (51, 75, 83). One study reported that home-made cooking is characteristic of the Eastern African diet, and one study, referring to all of Africa, mentioned fermentation (45).

### 3.6 Quality assessment

**Figure 3:**
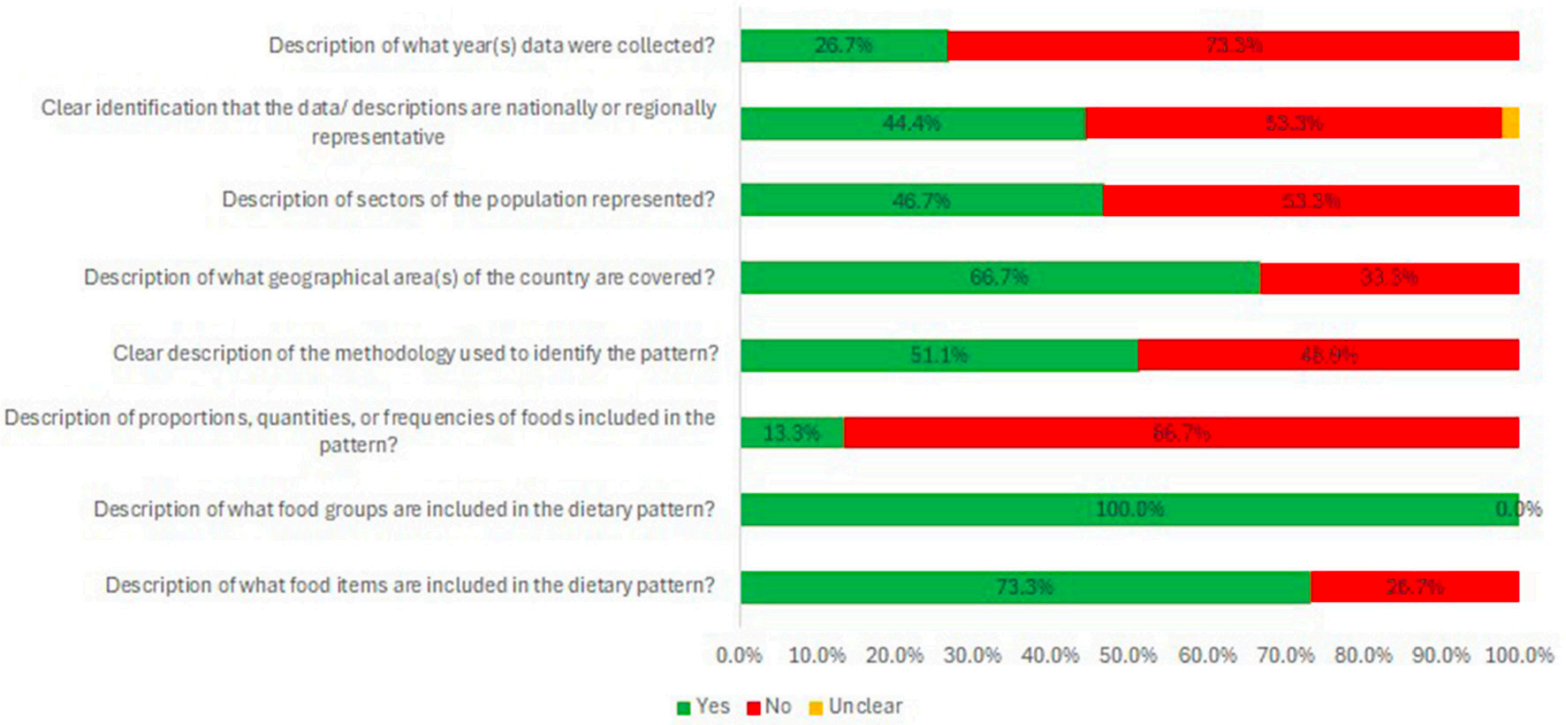
Quality assessment of reviewed studies.

Among the eight indicators of the quality assessment tool, only one, relating to the description of food groups, was reported by all studies (Figure 3 and Supplementary material I, Supplementary Table S11). Over 50% of the included studies reported the geographical areas, the methodology used to define the diet, and a description of food items. However, only a limited number of studies provided details on the proportions/quantities/frequency of consumption of the foods characterizing the TrAfDi and the years during which the data were collected (Figure 3).

## 4 Discussion

This study provides, to the best of our knowledge, the first evidenced description of the traditional African diet, systematically derived from the literature. Specifically, we set out to identify the food groups and food items consistently evidenced to be characteristic of the TrAfDi as a whole, highlighting regional differences. We found that maize, cassava (manioc), cowpeas (*vigna unguiculata*), fish, fruit, legumes, millet, sorghum, and vegetables were the most frequently cited food items (reported by >25% of the studies reviewed). The most frequently cited food groups (reported by >75% of the reviewed studies) were cereals and their products, pulses, seeds and nuts and their products, and vegetables and their products.

### 4.1 Definition of the TrAfDi

With >50% of all studies citing maize as a traditional food, the relevance of this food item to the TrAfDi across all regions of Africa cannot be disputed. With the other most highly cited food items being cassava (manioc), cowpeas (*vigna unguiculata*), fish, fruit, legumes, millet, sorghum, and vegetables, the continent-wide TrAfDi can be viewed as depending on a highly nutritious core of (mostly plant-based) foods providing carbohydrates, protein, healthy fats, vitamins, minerals, and fiber. This reinforces the view of traditional diets as dietary patterns that utilize locally sourced produce to provide health properties to surrounding communities, which have been tested over time (4, 6).

Analyses of the most cited food groups in different African regions indicate that there may be a low amount of regional variation in traditionally important foods, but this cannot be confirmed due to the imbalanced number of studies referring to different regions (Figure 2). The top five food groups cited by the collective analysis of all studies included were identical to those reported by studies referring to Eastern and Southern Africa, possibly because these comprised the majority of studies referring to specific regions (31 out of 43). These food groups comprise “cereals and their products,” “roots, tubers, plantains and their products,” “vegetables and their products,” “fruits and their products,” and “pulses, seeds and nuts and their products.” Studies referring to the whole of Africa differ slightly from this, with “meat and meat products” replacing “pulses, seeds and nuts and their products.” Western Africa differs in that, while “cereals and their products,” “roots, tubers, plantains and their products,” and “pulses, seeds and nuts and their products” feature in the top five cited food groups, “fats and oils” and “fish, shellfish and their products” also feature as characteristic of the traditional diet of this region. This may illustrate agro-ecological differences between Western Africa (largely tropical warm arid, semiarid, and subhumid), Eastern (more balanced tropical warm/cool arid, semiarid, and subhumid), and Southern Africa (a mix of subtropical warm/cool and tropical warm/cool) (90). Northern Africa was excluded from this regional analysis, as only one study referred specifically to this region (87). Similarly, Central Africa was not referred to by any of the studies included in our scoping review and, as such, did not feature in the regional diet analyses. While this scoping review was able to provide a generalized overview of traditional dietary patterns across the continent of Africa, the relatively few studies that referred to specific African regions and the exclusion of Northern and Central Africa from the regional analysis mean that these results should be considered as regionally indicative. Furthermore, the studies included did not permit further exploration of sub-regional dietary models or typologies that may relate to, or be derived from, Africa's vast cultural, agro-ecological, and culinary diversity. Nonetheless, these findings highlight the importance of regionally tailored nutritional interventions that account for region-specific traditional dietary patterns, particularly in Western Africa, while also underscoring the need for further research to confirm these observations, investigate the traditional diets of Northern and Central Africa, and put regional differences into more accurate region-specific cultural and agro-ecological contexts.

### 4.2 Food groups in the TrAfDi

The top five food groups with the highest numbers of food items reported to be characteristic of the TrAfDi, indicating the diversity of this dietary pattern, were (in descending order) “vegetables and their products” (122 items), “fruits and their products” (59 items), “pulses, seeds and nuts and their products” (51 items), “cereals and their products” (39 items), and “meat and meat products” (32 items). These highlight the vast diversity of plant-based foods from across the African continent, with four out of five traditional food groups being plant-derived, and point toward the health-giving potential of the TrAfDi. However, it should be noted that the methodological heterogeneity of studies included in this review may have biased these results.

Although benefit-exerting properties of the TrAfDi need to be established in further research, potential mechanisms through which this diet might be linked to health benefits include the high antioxidant and anti-inflammatory action of plant-based food products, supporting several metabolic functions, including the immune and circulatory systems (91). Similar to other studies of traditional diets, the predominant plant-based composition of the TrAfDi illustrates how healthy traditional food patterns can sustainably utilize naturally available food sources (6). This is relevant to needs and popular trends in present-day societies, as these traditional dietary practices can provide wider environmental benefits to climate, soil, and biodiversity, alongside the much sought-after health benefits (5).

Some studies reported indigenous foods and beverages, with 23 food items in total included in this food group, across all studies. These were mostly mentioned by studies referring to Southern Africa (28.6% of studies referring to that region). These items included porridges or drinks made primarily from ground cereals (often mixed with dairy products), soups with meats, seafood, and vegetables. However, given the inconsistent reporting of these food items throughout the reviewed studies and the sparse detail regarding ingredients, it is difficult to draw conclusions about how truly characteristic of the TrAfDi these items are. The food group “meat and meat products” would also be interesting to explore further, as in our scoping review it included food items such as African giant snail, game meat from wildlife, camel, and blood, alongside more agriculturalised meats, such as poultry, beef, lamb and pork. These items may reflect the close tie some communities have with wild areas, highlighting the procurement of foods from the natural environment in certain traditional foods systems (92). This also illustrates how foods and food groups capture expressions of region-specific culture and lifestyle (4).

### 4.3 Evolution of the TrAfDi over time

Many studies have suggested that the traditional diets in Africa have been significantly replaced or eliminated by colonization and neocolonial influences (55, 93, 94). Although these traditional practices were associated with various health benefits, they have been progressively replaced by a globalized food system dominated by multinational corporations (55). Given that only one study in this review reported on the TrAfDi before colonization, this review cannot give a full account of how the diet has changed over time. However, available evidence suggests substantial shifts in staple food consumption, particularly following the Columbian Exchange, the transcontinental movement of crops and goods that began after Columbus's voyages to the Americas.

Maize is one of the most prominent crops introduced to Africa after colonization, which aligns with findings from other studies. In the early stages of the agricultural era, the TrAfDi was characterized by wild vegetables, sorghum, finger millet, pearl millet, and yams (95, 96). After Columbus opened up the American continent to European exploration, the development of the Columbian Exchange had a profound influence on African foods (97, 98). This global exchange introduced several staple crops from the Americas, including maize, rice, peanuts (groundnuts), tomatoes, sweet potatoes, potatoes, kidney beans, pumpkins, and cassava (96, 99). Maize, in particular, spread widely across the African continent due to its high energy yield, low labor requirements, and short growing season (99). However, its expansion posed a threat to the continued use of indigenous crops, such as millet and sorghum (55). While this shift might have helped improve food availability in some regions, it might also have contributed to reduced dietary diversity and the decline of indigenous crops that were potentially more nutrient-dense and better suited to traditional food systems.

Cassava, another major crop introduced during the Columbian Exchange, became an important part of African diets because of its drought tolerance, adaptability to poor soils, and ability to be stored for long periods in processed forms, such as gari or fufu (100). According to a FAO report, cassava is a vital food security crop for millions of people, particularly in areas prone to food shortages (100). While it has contributed to food security in many African regions, reliance on cassava has also raised concerns due to its relatively low protein content compared to traditional grains, such as millet.

As only one study included in this review described dietary patterns before colonization, further research is needed to better understand how the TrAfDi has evolved and to identify the historical, social, and environmental forces that have shaped these dietary transitions. Nevertheless, our description of the TrAfDi after colonization remains relevant in understanding the potential health benefits of the TrAfDi amidst rising NCD rates, and could inform contemporary public health efforts that seek to preserve beneficial elements of this traditional diet amid ongoing dietary Westernization.

### 4.4 Amounts of food groups included in the TrAfDi

A limited number of studies (*n* = 4) provided information on food group quantities, which agrees with findings from recent systematic reviews that explored the definitions of the traditional Chinese (8) and Mexican (12) diets. Additionally, the reported quantities of food groups in the TrAfDi varied significantly across the literature, reflecting high heterogeneity. Despite almost all four studies reporting quantities for maize (banku), fish, and fruits, their level of reported consumption in the TrAfDi varied significantly (67, 71, 79, 80). Providing quantities of various food groups consumed is crucial when defining a dietary pattern; however, in the current review, the different methods utilized to describe food groups within the TrAfDi led to heterogeneous outcomes, hindering comparisons. For instance, one study reported food group consumption using the percentage of total energy intake, which depends on the energy density of the analyzed food groups. This approach suggests that foods with lower energy densities, such as rice and maize, appear to contribute less to the diet, even when they are consumed frequently or in large amounts, and differs from the findings of studies that reported food group quantities using average daily consumption (67, 80). Such inconsistencies complicate efforts to accurately determine the quantities of food groups in the TrAfDi. Moreover, variations in how food groups are categorized could influence outcomes. Considering the limited available data on the amounts of food groups that are characteristic of the TrAfDi and the high heterogeneity among studies, a more precise and quantitative description of the TrAfDi is essential. Future research should therefore focus on exploring and standardizing food group quantities within this dietary pattern.

### 4.5 Strengths and limitations

To the best of our knowledge, this is the first study to systematically scope the existing evidence on the definition of the TrAfDi, incorporating both peer-reviewed and gray literature without restrictions on time frame or geographical location. Considering that eating habits can change significantly over time, exploring the definition of a country's traditional dietary patterns by, e.g., relying solely on interviews with elderly populations, as used in previous studies (101), may not be suitable. Instead, analyzing the frequency of citations provides a more comprehensive and less biased perspective on the food groups and food items that define the TrAfDi. Additionally, variations in the TrAfDi were explored to highlight potential regional differences in this dietary pattern. Beyond nutritional perspectives, the cultural, historical, and agricultural significance of the TrAfDi was also examined, emphasizing how dietary patterns are integrated with lifestyle factors. Furthermore, this article focuses on exploring the definition of the TrAfrDi; however, the methods utilized can be adapted by researchers interested in defining other traditional dietary patterns globally.

There are some limitations to this review. First, only a small number of studies were available for certain regions, which limited the ability to establish a clear definition of the TrAfDi in those areas. This highlights the need for further research into regional African diets. Most of the reviewed studies focused on countries in Eastern, Western, and Southern Africa, which means the proposed definition of the TrAfDi may disproportionately reflect dietary patterns from these regions, particularly those in Southern Africa. However, as a scoping review, the findings are based on the currently available literature, and this geographic imbalance highlights the need for more research in underrepresented areas, particularly Northern and Central Africa. In addition, characterizing the traditional diet of an area can be inherently complex. Dietary components and eating patterns can vary, not only between countries but also within regions of the same country. These differences are further influenced by factors such as the availability of specific food groups, economic and industrial development, environmental change, and shifts in agricultural practices. These contextual variations make it challenging to define a single, cohesive traditional diet for any given area. Second, the included studies employed varying methods to define the TrAfDi, with the majority relying on a posteriori approaches and selecting food groups based on previous literature. While a posteriori methods are important in reflecting current dietary patterns, they may not accurately represent a “traditional” diet (102). Future studies investigating the TrAfDi should therefore consider utilizing a priori approaches to define this dietary pattern, particularly focusing on each region's diet when assessing links between adherence to the TrAfDi and health or environmental outcomes (8). As only one study focused on the TrAfDi before colonization, further research is needed to determine whether the food groups characterizing the TrAfDi have remained consistent over time.

Considering that the African continent is the second largest continent in the world, with an exceptionally wide range of climate zones and remarkable cultural, ethnic, and linguistic diversity (103), the TrAfDi is likely to vary substantially across regions, cultural groups, and geopolitical boundaries. Notably, traditional food systems do not necessarily align with modern political borders, as many cultural and ethnic communities span multiple countries (103). For instance, the traditional diets of the Maasai in Kenya and the Igbo in Nigeria show notable differences between each other, reflecting distinct cultural, environmental, and historical influences (104). However, the ongoing nutrition transition in Africa has increasingly shifted traditional diets toward more Westernized eating patterns, which are associated with a higher risk of NCDs (105, 106). Given this context, this study aimed to summarize the common characteristics of the TrAfDi by identifying the most frequently mentioned food groups in the available literature at a continental level. This approach helps to develop an overarching definition that reflects broad dietary patterns shared across diverse African communities. Establishing such a definition is important for informing continent-wide public health strategies, while remaining sensitive to the cultural and ecological diversity that exists within and across countries.

Additionally, food groups reported to characterize the TrAfDi in the reviewed studies may reflect the researchers' choices, and not necessarily the true definition of this dietary pattern. Changes in food groups that characterize the TrAfDi should also be further explored to identify whether the food groups are consistently “traditional” (based on historical continuity, cultural relevance, and pre-colonial origins) or influenced by the current diet. Future research is essential to develop standardized methodologies for defining the TrAfDi, ensuring a more accurate and holistic understanding of its characteristics, cultural, historical, and agricultural relevance, and its implications for health and sustainability. Finally, potentially eligible papers might have been overlooked due to the specific inclusion and exclusion criteria of the current review and the specific databases searched, an inherent limitation of any review paper. Future studies could widen the search strategy for eligible papers by using additional databases, such as Scopus.

## 5 Conclusion

The TrAfDi is a diverse, plant-rich traditional food system. It does not appear to vary greatly at the food group level between African regions, although further research is required to confirm this. As reporting of food group quantities and methods of preparation was minimal in the reviewed studies, there is an additional need for studies that integrate cultural knowledge to standardize dietary intake.

To the best of our knowledge, no systematically derived and/or consistent characterization of the TrAfDi exists to date, and, as such, this study provides crucial initial insights into the traditional dietary patterns of the African continent. Our findings are therefore important for understanding the cultural implications of shifting availability of region-specific traditional food groups in a changing climate, accompanied by economic transitions, as well as identifying culturally-acceptable and nutritionally important food groups that should be promoted to enhance food security efforts in a climate-altered Africa. Findings of this review can also help promote the consumption of healthy and culturally-relevant traditional diets, particularly in African regions with rising obesity and NCD rates. This would be essential to support the development of policies on sustainable healthcare, as culturally relevant dietary patterns can deliver effective, flexible health and wellness practices that can be easily adopted (107). Given the high proportion of plant-based foods in the TrAfDi, this may additionally provide incentives to invest in enhanced plant health management in African communities most vulnerable to the impacts of climate change.

## Data Availability

The original contributions presented in the study are included in the article/Supplementary material, further inquiries can be directed to the corresponding author.
