# Supplementary material for: Definition of the traditional African diet: a scoping review
Source: Front Nutr. 2025 Sep 12;12:1651945. doi: 10.3389/fnut.2025.1651945 (PMC12465632; doi:10.3389/fnut.2025.1651945)
Supplement: Supplementary file 1 [file Data_Sheet_1.zip › Supplementary Material I.docx]

# Supplementary Material I

**Table S1. PRISMA-ScR Checklist.**

| **SECTION** | **ITEM** | **PRISMA-ScR CHECKLIST ITEM** | **REPORTED IN SECTION #** |
| --- | --- | --- | --- |
| **TITLE** | | | |
| Title | 1 | Identify the report as a scoping review. | Title and 2.1 Study design |
| **ABSTRACT** | | | |
| Structured summary | 2 | Provide a structured summary that includes (as applicable): background, objectives, eligibility criteria, sources of evidence, charting methods, results, and conclusions that relate to the review questions and objectives. | Abstract section |
| **INTRODUCTION** | | | |
| Rationale | 3 | Describe the rationale for the review in the context of what is already known. Explain why the review questions/objectives lend themselves to a scoping review approach. | 1. Introduction |
| Objectives | 4 | Provide an explicit statement of the questions and objectives being addressed with reference to their key elements (e.g., population or participants, concepts, and context) or other relevant key elements used to conceptualize the review questions and/or objectives. | 1. Introduction |
| **METHODS** | | | |
| Protocol and registration | 5 | Indicate whether a review protocol exists; state if and where it can be accessed (e.g., a Web address); and if available, provide registration information, including the registration number. | 2.1 Study design |
| Eligibility criteria | 6 | Specify characteristics of the sources of evidence used as eligibility criteria (e.g., years considered, language, and publication status), and provide a rationale. | 2.3 Study selection |
| Information sources* | 7 | Describe all information sources in the search (e.g., databases with dates of coverage and contact with authors to identify additional sources), as well as the date the most recent search was executed. | Supplementary material I |
| Search | 8 | Present the full electronic search strategy for at least 1 database, including any limits used, such that it could be repeated. | 2.2 Identifying relevant studies and Supplementary material I |
| Selection of sources of evidence† | 9 | State the process for selecting sources of evidence (i.e., screening and eligibility) included in the scoping review. | 2.3 Study selection |
| Data charting process‡ | 10 | Describe the methods of charting data from the included sources of evidence (e.g., calibrated forms or forms that have been tested by the team before their use, and whether data charting was done independently or in duplicate) and any processes for obtaining and confirming data from investigators. | 2.4 Charting the data and quality assessment |
| Data items | 11 | List and define all variables for which data were sought and any assumptions and simplifications made. | 2.5 Collating, summarising, and reporting the results |
| Critical appraisal of individual sources of evidence§ | 12 | If done, provide a rationale for conducting a critical appraisal of included sources of evidence; describe the methods used and how this information was used in any data synthesis (if appropriate). | 2.4 Charting the data and quality assessment |
| Synthesis of results | 13 | Describe the methods of handling and summarizing the data that were charted. | 2.5 Collating, summarising, and reporting the results |
| **RESULTS** | | | |
| Selection of sources of evidence | 14 | Give numbers of sources of evidence screened, assessed for eligibility, and included in the review, with reasons for exclusions at each stage, ideally using a flow diagram. | 3. Results and Figure 1 |
| Characteristics of sources of evidence | 15 | For each source of evidence, present characteristics for which data were charted and provide the citations. | 3.1 Study characteristics |
| Critical appraisal within sources of evidence | 16 | If done, present data on critical appraisal of included sources of evidence (see item 12). | 3.6 Quality assessment and Figure 2 |
| Results of individual sources of evidence | 17 | For each included source of evidence, present the relevant data that were charted that relate to the review questions and objectives. | Section 3.2 to 3.5, Supplementary material II |
| Synthesis of results | 18 | Summarize and/or present the charting results as they relate to the review questions and objectives. | Section 3.2 to 3.5, Supplementary material II |
| **DISCUSSION** | | | |
| Summary of evidence | 19 | Summarize the main results (including an overview of concepts, themes, and types of evidence available), link to the review questions and objectives, and consider the relevance to key groups. | Section 4.1 to 4.4 |
| Limitations | 20 | Discuss the limitations of the scoping review process. | 4.5 Strengths and limitations |
| Conclusions | 21 | Provide a general interpretation of the results with respect to the review questions and objectives, as well as potential implications and/or next steps. | 5. Conclusions |
| **FUNDING** | | | |
| Funding | 22 | Describe sources of funding for the included sources of evidence, as well as sources of funding for the scoping review. Describe the role of the funders of the scoping review. | Funding section |

JBI = Joanna Briggs Institute; PRISMA-ScR = Preferred Reporting Items for Systematic reviews and Meta-Analyses extension for Scoping Reviews.

* Where *sources of evidence* (see second footnote) are compiled from, such as bibliographic databases, social media platforms, and Web sites.

† A more inclusive/heterogeneous term used to account for the different types of evidence or data sources (e.g., quantitative and/or qualitative research, expert opinion, and policy documents) that may be eligible in a scoping review as opposed to only studies. This is not to be confused with *information sources* (see first footnote).

‡ The frameworks by Arksey and O’Malley (6) and Levac and colleagues (7) and the JBI guidance (4, 5) refer to the process of data extraction in a scoping review as data charting*.*

§ The process of systematically examining research evidence to assess its validity, results, and relevance before using it to inform a decision. This term is used for items 12 and 19 instead of "risk of bias" (which is more applicable to systematic reviews of interventions) to include and acknowledge the various sources of evidence that may be used in a scoping review (e.g., quantitative and/or qualitative research, expert opinion, and policy document).

*From:* Tricco AC, Lillie E, Zarin W, O'Brien KK, Colquhoun H, Levac D, et al. PRISMA Extension for Scoping Reviews (PRISMAScR): Checklist and Explanation. Ann Intern Med. 2018;169:467–473. [doi: 10.7326/M18-0850](http://annals.org/aim/fullarticle/2700389/prisma-extension-scoping-reviews-prisma-scr-checklist-explanation).

**Table S2. Search strategy.**

**African Journal Archive**

tradition* Africa* diet*/ tradition* Africa* eat*

***Total articles retrieved: 7679***

**SciELO Citation Index on Web of Science**

tradition* OR region* OR nation* OR native OR indigenous

AND

Africa*

AND

diet* OR “diet* pattern*” OR “eating pattern*” OR “food habit*” OR “eating habit*” OR “diet* habit*” OR cuisine

***Total articles retrieved: 77***

**International Bibliography of the Social Sciences (IBSS)**

Summary (Africa*)

AND

summary(tradition* OR region* OR nation* OR native OR indigenous)

AND

summary(diet* or "diet* pattern*" or "food pattern*" or "eating pattern*" or "food habit*" or "eating habit*" or "diet* habit" or cuisine

***Total articles retrieved: 226***

**Web of Science Core Collection**

Africa*

AND

tradition* OR region* OR nation* OR native OR indigenous

AND

diet* or "diet* pattern*" or "food pattern*" or "eating pattern*" or "food habit*" or "eating habit*" or "diet* habit" or cuisine

***Total articles retrieved: 5060***

**PubMed**

((Africa*[Title/Abstract])

AND

(tradition*[Title/Abstract] OR region*[Title/Abstract] OR nation*[Title/Abstract] OR native[Title/Abstract] OR indigenous[Title/Abstract])) AND (diet*[Title/Abstract] OR "diet* pattern*"[Title/Abstract] OR "food pattern*"[Title/Abstract] OR "eating pattern*"[Title/Abstract] OR "food habit*"[Title/Abstract] OR "eating habit*"[Title/Abstract] OR "diet habit*"[Title/Abstract] OR cuisine[Title/Abstract])

***Total articles retrieved: 2474***

**CENTRAL and Cochrane Reviews**

Africa*

AND

tradition* OR region* OR nation* OR native OR indigenous

AND

diet* or "diet* pattern*" or "food pattern*" or "eating pattern*" or "food habit*" or "eating habit*" or "diet* habit" or cuisine

***Total articles retrieved in Cochrane Reviews: 10***

***Total articles retrieved in Trials: 291***

***Total articles retrieved in editorials: 1***

**Table S3. Data extraction form.**

| Study ID |
| --- |
| Author name and article title |
| Year of publication |
| Country |
| Characteristics of population, including age (mean/SD), number of participants, sex, ethnicity |
| Study design |
| Year/ period represented |
| Geographical location represented |
| Description of traditional African diet |
| Food items contained |
| Food groups contained |
| Quantity of food items/ food groups |
| Cooking methods involved |
| Methods of identification of diet |
| Diet collection method |

ID, identification; SD, standard deviation.

**Table S4. Quality assessment of included reports*.**

- Report describes the food items included in the dietary pattern (1 for yes, 0 for no)
- Report describes the food groups included in the dietary pattern (1 for yes, 0 for no)
- Report describes the proportions, quantities, or frequencies of foods included in the dietary pattern (1 for yes, 0 for no)
- Report clearly describes the methodology used to identify the dietary pattern (1 for yes, 0 for no)
- Report describes what geographical area(s) of the continent/ country are covered (1 for yes, 0 for no)
- Report describes the population(s) represented (1 for yes, 0 for no)
- There is a clear identification of the population(s) as located in Africa or being of African origin/ancestry (1 for yes, 0 for no)
- Report describes the year(s) data were collected (1 for yes, 0 for no)

* Adapted from:

Green, R., J. Milner, E. J. Joy, S. Agrawal and A. D. Dangour (2016). "Dietary patterns in India: a systematic review." Br J Nutr **116**(1): 142-148

Valerino-Perea, S., L. Lara-Castor, M. E. G. Armstrong and A. Papadaki (2019). "Definition of the Traditional Mexican Diet and Its Role in Health: A Systematic Review." Nutrients **11**(11): 2803

**Table S7: Food items and groups.**

| **Food item** | **Food group** |
| --- | --- |
| Abelmoschus esculentus | Vegetables and their products |
| Ackee | Fruits and their products |
| African breadfruit (Treculia africana) | Fruits and their products |
| African Giant snail (Archachatina marginata) | Meat and meat products |
| African locust bean (Parkia biglobosa) | Pulses, seeds and nuts and their products |
| African nutmeg | Spices and condiments |
| African oil bean (Pentaclethra tnacropltylla) | Pulses, seeds and nuts and their products |
| African pear (Dacryodes edulis) | Fruits and their products |
| African rice (Oryza glaberrima) | Cereals and their products |
| African walnut (Coula edulis) | Pulses, seeds and nuts and their products |
| African yam (Dioscorea rotundata) | Roots, tubers, plantains and their products |
| African yam bean | Pulses, seeds and nuts and their products |
| Agaricus species | Vegetables and their products |
| Agushie (pumpkin seeds) | Vegetables and their products |
| Amaranth | Vegetables and their products |
| Amaranth leaf | Vegetables and their products |
| Amaranthus thumbergii | Vegetables and their products |
| Animal foods | Meat and meat products |
| Animal products | Meat and meat products |
| Animals, wild | Meat and meat products |
| Ants | Insects, grubs and their products |
| Apple | Fruits and their products |
| Artemisia | Foods for particular nutritional uses |
| Auricularia auricular Judae (Bull) Quél | Vegetables and their products |
| Avocado | Vegetables and their products |
| Bambara groundnuts (Vigna sub-terranea) | Pulses, seeds and nuts and their products |
| Bananas | Fruits and their products |
| Bananas, sweet | Fruits and their products |
| Bananas, yellow | Fruits and their products |
| Banku | Roots, tubers, plantains and their products |
| Baobab (Adansonia digitata) | Vegetables and their products |
| Baobab leaves | Vegetables and their products |
| Barley | Cereals and their products |
| Bean or pumpkin-leaf | Vegetables and their products |
| Beans | Pulses, seeds and nuts and their products |
| Beef | Meat and meat products |
| Beef, cooked (seswaa) | Meat and meat products |
| Beef, dried (biltong) | Meat and meat products |
| Beetroot | Vegetables and their products |
| Berries | Fruits and their products |
| Beverages | Beverages |
| Birds | Meat and meat products |
| Biscuits | Composite dishes |
| Bitter gourd | Vegetables and their products |
| Bitter leaf (Vernonia amygdalina) | Vegetables and their products |
| Bitterberries | Vegetables and their products |
| Black bean | Pulses, seeds and nuts and their products |
| Blackberry | Fruits and their products |
| Blackjack (Bidens pilosa) | Vegetables and their products |
| Blood | Meat and meat products |
| Bogobe | Indigenous dishes or beverages or beverages |
| Bottle gourds | Vegetables and their products |
| Bran flakes | Cereals and their products |
| Bread, white or dark | Cereals and their products |
| Brown or white beans | Pulses, seeds and nuts and their products |
| Brukina | Indigenous dishes or beverages or beverages |
| Buhunda | Fruits and their products |
| Buns | Cereals and their products |
| Burukutu | Indigenous dishes or beverages or beverages |
| Bushveld cherry (Pappea capensis) | Fruits and their products |
| Butternut squash | Vegetables and their products |
| Buyeko | Fruits and their products |
| Cabbage, cooked | Vegetables and their products |
| Cabbage, Ethiopian (Brassica carinata) | Vegetables and their products |
| Cabbage, European | Vegetables and their products |
| Cabbage, fresh | Vegetables and their products |
| Cactus pear | Fruits and their products |
| Cajanus cajan | Pulses, seeds and nuts and their products |
| Caju | Pulses, seeds and nuts and their products |
| Camel | Meat and meat products |
| Cape fig (Ficus sur) | Fruits and their products |
| Cape holly (Ilex mitis) | Fruits and their products |
| Carbohydrates, unrefined | Cereals and their products |
| Carissa grandflora | Fruits and their products |
| Carrot | Vegetables and their products |
| Cashew | Pulses, seeds and nuts and their products |
| Cassava (manioc) | Roots, tubers, plantains and their products |
| Cassava leaves | Vegetables and their products |
| Castor oil seeds | Pulses, seeds and nuts and their products |
| Caterpillars | Insects, grubs and their products |
| Celosia argentea | Vegetables and their products |
| Cereal grains | Cereals and their products |
| Cereals, wholegrain | Cereals and their products |
| Chakata | Fruits and their products |
| Chard | Vegetables and their products |
| Chicken | Meat and meat products |
| Cleome gynandra | Vegetables and their products |
| Cluster beans | Pulses, seeds and nuts and their products |
| Coconut | Pulses, seeds and nuts and their products |
| Cocoyam | Roots, tubers, plantains and their products |
| Coleus potato | Roots, tubers, plantains and their products |
| Collybia butyracea (Bull) P Kumm | Vegetables and their products |
| Coprinus atramentarius (Bull) Fr | Vegetables and their products |
| Coprinus picaceus (Bull) Gray | Vegetables and their products |
| Corchorus olitorius | Vegetables and their products |
| Corchorus tridens | Vegetables and their products |
| Corchorus trilocularis | Vegetables and their products |
| Cordyceps sinensis | Vegetables and their products |
| Corn flakes | Cereals and their products |
| Couscous | Cereals and their products |
| Cow liver | Meat and meat products |
| Cowpea leaves (Morogo wa dinawa) | Pulses, seeds and nuts and their products |
| Cowpeas (Vigna unguiculata) | Pulses, seeds and nuts and their products |
| Crab | Fish, shellfish and their products |
| Cucumber | Vegetables and their products |
| Cucumber, wild | Vegetables and their products |
| Cucumis hirsutus | Vegetables and their products |
| Curcumin | Spices and condiments |
| Daedaleopsis confragosa | Vegetables and their products |
| Dairy products | Milk and milk products |
| Dates | Fruits and their products |
| Deep-fried wheat flour dough | Indigenous dishes or beverages or beverages |
| Derere (okra) | Vegetables and their products |
| Desert mushroom | Vegetables and their products |
| Devil's thorn (Sesamum eriocarpum) | Vegetables and their products |
| Dika | Pulses, seeds and nuts and their products |
| Dolo | Indigenous dishes or beverages or beverages |
| Edible insects including grasshoppers locusts crickets beetles caterpillars bees ants grasshoppers locusts crickets and termites | Insects, grubs and their products |
| Eggplant | Vegetables and their products |
| Eggs | Eggs and their products |
| Egusi | Indigenous dishes or beverages |
| Eland's wattle (Elephantorrhiza elephantina) | Pulses, seeds and nuts and their products |
| Ensete (Ensete vantricosum) | Fruits and their products |
| False yam | Roots, tubers, plantains and their products |
| Fat hen (Chenopodium album) | Vegetables and their products |
| Fats | Fats and oils |
| Fermented beverages | Beverages |
| Fermented foods | Foods for particular nutritional uses |
| Figs | Fruits and their products |
| Fish | Fish, shellfish and their products |
| Fish, dried | Fish, shellfish and their products |
| Fluted pumpkin | Vegetables and their products |
| Fonio (Digitaria exilis) | Cereals and their products |
| Fried foods | Composite dishes |
| Fruit | Fruits and their products |
| Fruit juice | Fruits and their products |
| Fruit, tropical | Fruits and their products |
| Fruit, wild | Fruits and their products |
| Fufu | Indigenous dishes or beverages or beverages |
| Fura de nunu | Indigenous dishes or beverages or beverages |
| Ganoderma lucidum | Vegetables and their products |
| Garden egg (African eggplant) | Vegetables and their products |
| Garlic | Vegetables and their products |
| Geocarpa groundnut (Macrotyloma geocarpum) | Pulses, seeds and nuts and their products |
| Ginger | Spices and condiments |
| Goat | Meat and meat products |
| Gourd | Vegetables and their products |
| Gowé | Indigenous dishes or beverages or beverages |
| Grains | Cereals and their products |
| Grasshoppers | Insects, grubs and their products |
| Green leafy vegetables | Vegetables and their products |
| Green tea | Beverages |
| Green vegetables | Vegetables and their products |
| Greens | Vegetables and their products |
| Grifola frondosa | Vegetables and their products |
| Groundnut porridge | Pulses, seeds and nuts and their products |
| Groundnuts (Arachis hypogaea) | Pulses, seeds and nuts and their products |
| Guava | Fruits and their products |
| Guinea fowl | Meat and meat products |
| Guinea yam | Roots, tubers, plantains and their products |
| Gymnanthemum amygdalinum | Vegetables and their products |
| Herbs | Vegetables and their products |
| Herbs, wild | Vegetables and their products |
| Hericium erinaceus | Vegetables and their products |
| Hibiscus (Hibiscus sabdariffa) | Vegetables and their products |
| Honey | Sweets and sugars |
| Horseradish tree leaves | Vegetables and their products |
| Hyacinth beans | Pulses, seeds and nuts and their products |
| Indigenous fruit | Fruits and their products |
| Indigenous plant foods | Vegetables and their products |
| Indigenous vegetables | Vegetables and their products |
| Insects | Insects, grubs and their products |
| Isijabane | Indigenous dishes or beverages |
| Isijingi | Indigenous dishes or beverages |
| Isiphuphutho | Indigenous dishes or beverages |
| Jute mallow | Vegetables and their products |
| Kaffir beer | Beverages |
| Kaffir corn | Cereals and their products |
| Kaffir melon | Fruits and their products |
| Kaffir potato (Coleus esculentus) | Roots, tubers, plantains and their products |
| Kale | Vegetables and their products |
| Kenkey | Indigenous dishes or beverages |
| Kenkiliba leaves | Vegetables and their products |
| Kidney beans | Pulses, seeds and nuts and their products |
| Kola | Pulses, seeds and nuts and their products |
| Koobo berry | Fruits and their products |
| Kunun-Zaki | Indigenous dishes or beverages or beverages |
| Lactarius trivialis Fr | Vegetables and their products |
| Lagneria vulgaris | Vegetables and their products |
| Lamb | Meat and meat products |
| Langbeen bossie (rooibos) | Beverages |
| Lard | Fats and oils |
| Laricifomes officinalis | Vegetables and their products |
| Legumes | Pulses, seeds and nuts and their products |
| Lemons | Fruits and their products |
| Lemons, unripe | Fruits and their products |
| Lentils | Pulses, seeds and nuts and their products |
| Lentinula edodes | Vegetables and their products |
| Lentinus squarrosulus Mont | Vegetables and their products |
| Lenzites betulina | Vegetables and their products |
| Lerotse | Fruits and their products |
| Lettuce | Vegetables and their products |
| Lima beans | Pulses, seeds and nuts and their products |
| Limes | Fruits and their products |
| Liver | Meat and meat products |
| Local cereals | Cereals and their products |
| Local roots | Roots, tubers, plantains and their products |
| Locusts | Insects, grubs and their products |
| Macademia | Pulses, seeds and nuts and their products |
| Macrolepiota species | Vegetables and their products |
| Madora | Insects, grubs and their products |
| Mahewu | Indigenous dishes or beverages or beverages |
| Maize | Cereals and their products |
| Maize (white and yellow) | Cereals and their products |
| Maize kernel | Cereals and their products |
| Maize meal | Cereals and their products |
| Maize products, fermented | Cereals and their products |
| Mamonyi | Fruits and their products |
| Mande | Fruits and their products |
| Mangoes | Fruits and their products |
| Margarine | Fats and oils |
| Marula (seasonal) | Fruits and their products |
| Matohwe | Fruits and their products |
| Mazhanje | Fruits and their products |
| Mboco | Pulses, seeds and nuts and their products |
| Mealie | Cereals and their products |
| Mealie meal, stone ground | Cereals and their products |
| Mealie rice | Cereals and their products |
| Mealies (maize), green or dried | Cereals and their products |
| Mealies, stamp | Cereals and their products |
| Mealies, whole | Cereals and their products |
| Meat | Meat and meat products |
| Meat, game from wildlife | Meat and meat products |
| Meat, processed | Meat and meat products |
| Meat, red | Meat and meat products |
| Meat, salted | Meat and meat products |
| Meat, traditional | Meat and meat products |
| Meat, white | Meat and meat products |
| Melon | Fruits and their products |
| Melon seed (Cucumeropsis mannii and Citrullus lanatus) | Fruits and their products |
| Melon, wild | Fruits and their products |
| Mfiru | Fruits and their products |
| Mice | Meat and meat products |
| Milk | Milk and milk products |
| Milk (full cream) | Milk and milk products |
| Milk products | Milk and milk products |
| Milk, cow | Milk and milk products |
| Milk, fermented | Milk and milk products |
| Milk, fresh | Milk and milk products |
| Milk, goat | Milk and milk products |
| Milk, raw cow | Milk and milk products |
| Milk, sour | Milk and milk products |
| Millet | Cereals and their products |
| Millet flour | Cereals and their products |
| Millet, finger | Cereals and their products |
| Mopane worms | Insects, grubs and their products |
| Moringa | Vegetables and their products |
| Mormodica balsamina | Vegetables and their products |
| Mung beans | Pulses, seeds and nuts and their products |
| Mushroom | Vegetables and their products |
| Mutton | Meat and meat products |
| Native pototo | Roots, tubers, plantains and their products |
| Ndobe | Fruits and their products |
| Nerè | Vegetables and their products |
| Néré seeds | Pulses, seeds and nuts and their products |
| Niébé | Pulses, seeds and nuts and their products |
| Nightshade, African (Solanum species) | Vegetables and their products |
| Nightshade, Black (Solanum nigrum) | Vegetables and their products |
| Nshima | Indigenous dishes or beverages |
| Nuts | Pulses, seeds and nuts and their products |
| Oats | Cereals and their products |
| Offal | Meat and meat products |
| Ogi | Indigenous dishes or beverages or beverages |
| Oil palm crops | Fats and oils |
| Oil seeds (Rvingia gabonensis var excelsa) | Pulses, seeds and nuts and their products |
| Oils | Fats and oils |
| Okra | Vegetables and their products |
| Olive oil | Fats and oils |
| Olives | Vegetables and their products |
| Olives, wild | Vegetables and their products |
| Onion | Vegetables and their products |
| Oranges | Fruits and their products |
| Oriolus versicolor | Vegetables and their products |
| Palm oil | Fats and oils |
| Papaya | Fruits and their products |
| Passion fruit | Fruits and their products |
| Pasta | Composite dishes |
| Pastries | Composite dishes |
| Pawpaw | Fruits and their products |
| Pearl millet | Cereals and their products |
| Peas | Pulses, seeds and nuts and their products |
| Pecan nuts | Pulses, seeds and nuts and their products |
| Pepper | Vegetables and their products |
| Pepper (green/red/yellow) | Vegetables and their products |
| Phaletshe or papa | Cereals and their products |
| Pigeon | Meat and meat products |
| Pigeon peas (Cajanus cajan) | Pulses, seeds and nuts and their products |
| Pineapples | Fruits and their products |
| Pizza | Composite dishes |
| Plant foods | Vegetables and their products |
| Plantain | Roots, tubers, plantains and their products |
| Plants, wild | Vegetables and their products |
| Pleurotus ostreatus | Vegetables and their products |
| Pleurotus pulmonarius | Vegetables and their products |
| Plum, wild | Fruits and their products |
| Pods | Pulses, seeds and nuts and their products |
| Polony | Roots, tubers, plantains and their products |
| Pork | Meat and meat products |
| Porridge, maize | Cereals and their products |
| Porridge, soft made with sorghum | Cereals and their products |
| Porridges, starchy | Cereals and their products |
| Potato, English | Roots, tubers, plantains and their products |
| Potato, native | Roots, tubers, plantains and their products |
| Potatoes | Roots, tubers, plantains and their products |
| Poultry | Meat and meat products |
| Prickly pear | Fruits and their products |
| Prosopis africana seeds | Pulses, seeds and nuts and their products |
| Psathyrella atroumbonata Pegler | Vegetables and their products |
| Pulses | Pulses, seeds and nuts and their products |
| Pumpkin | Vegetables and their products |
| Pumpkin bean | Pulses, seeds and nuts and their products |
| Purslane | Vegetables and their products |
| Rabbit | Meat and meat products |
| Rape (chomolia) | Vegetables and their products |
| Rape greens | Vegetables and their products |
| Red bean (kunde) | Pulses, seeds and nuts and their products |
| Red sorrel | Vegetables and their products |
| Rice | Cereals and their products |
| Rice, white | Cereals and their products |
| Rooibos | Vegetables and their products |
| Roots | Roots, tubers, plantains and their products |
| Roselle (Hibiscus sabdariffa) | Vegetables and their products |
| Rozites caperata | Vegetables and their products |
| Sadza | Indigenous dishes or beverages or beverages |
| Samosa | Composite dishes |
| Schizophyllum commune Fr | Vegetables and their products |
| Sclerotium-forming Pleurotus tuberregium | Vegetables and their products |
| Seafood | Fish, shellfish and their products |
| Seaweed | Vegetables and their products |
| Seeds | Pulses, seeds and nuts and their products |
| Senna leaves | Vegetables and their products |
| Serobe (offal) | Meat and meat products |
| Sesame | Pulses, seeds and nuts and their products |
| Shea butter fruit | Pulses, seeds and nuts and their products |
| Sheep | Meat and meat products |
| Shellfish | Fish, shellfish and their products |
| Small green pea (thuu) | Pulses, seeds and nuts and their products |
| Small peas (thoroko or chirok) | Pulses, seeds and nuts and their products |
| Small round green pea (podzo) | Pulses, seeds and nuts and their products |
| Soap aloe (Aloe maculata) | Vegetables and their products |
| Solanum macrocarpon | Vegetables and their products |
| Sorghum | Cereals and their products |
| Sorghum, sweet reed | Cereals and their products |
| Soumbalà | Indigenous dishes or beverages or beverages |
| Soups | Composite dishes |
| Sour plum | Fruits and their products |
| Soy beans | Pulses, seeds and nuts and their products |
| Soy milk | Pulses, seeds and nuts and their products |
| Spices | Spices and condiments |
| Spider whips | Insects, grubs and their products |
| Spiderplant (Basella alba) | Vegetables and their products |
| Spinach | Vegetables and their products |
| Squash | Vegetables and their products |
| Starchy roots | Roots, tubers, plantains and their products |
| Starchy staples | Foods for particular nutritional uses |
| Stews | Composite dishes |
| Sugar | Sweets and sugars |
| Sugar cane | Sweets and sugars |
| Sumuka wiki | Vegetables and their products |
| Sungwa | Fruits and their products |
| Sweet potato | Roots, tubers, plantains and their products |
| Sweet potato leaves | Vegetables and their products |
| Tamarind tree leaves | Vegetables and their products |
| Tamarinds | Fruits and their products |
| Tangerines | Fruits and their products |
| Tannia (Xanthosoma sagittifolium) | Roots, tubers, plantains and their products |
| Taro (Colacasia esculenta) | Roots, tubers, plantains and their products |
| Tea | Beverages |
| Tea with fat cakes | Beverages |
| Teff (Eragrotis tef (Zucc) Trotter | Cereals and their products |
| Termites | Insects, grubs and their products |
| Termitomyces clypeatus Heim | Vegetables and their products |
| Termitomyces globules | Vegetables and their products |
| Termitomyces globules Heim and Gooss | Vegetables and their products |
| Termitomyces mammiformis Heim | Vegetables and their products |
| Termitomyces microcarpus (Berk and Br) Heim | Vegetables and their products |
| Termitomyces robustus (Beeli) Heim | Vegetables and their products |
| Thistle, wild | Vegetables and their products |
| Tiger nuts (yellow nutsedge) | Roots, tubers, plantains and their products |
| Tô | Indigenous dishes or beverages or beverages |
| Tomatoes | Fruits and their products |
| Tree nuts | Pulses, seeds and nuts and their products |
| Tree tomatoes | Fruits and their products |
| Tricholoma species | Vegetables and their products |
| Truffles | Vegetables and their products |
| Tubers | Roots, tubers, plantains and their products |
| Turkey | Meat and meat products |
| Umnqutsho | Indigenous dishes or beverages |
| Vegetable oils | Fats and oils |
| Vegetables | Vegetables and their products |
| Vegetables, salad | Vegetables and their products |
| Vegetables, wild | Vegetables and their products |
| Velvet tamarind (Dialium guineense) | Fruits and their products |
| Volvariella esculenta | Vegetables and their products |
| Volvariella volvacea (Bull) Singer | Vegetables and their products |
| Wanderer's food | Indigenous dishes or beverages |
| Watermelon | Fruits and their products |
| Watermelon, wild | Fruits and their products |
| Wheat | Cereals and their products |
| Wild medlar (Vangueria infausta) | Fruits and their products |
| Wine | Beverages |
| Wood sorrel | Roots, tubers, plantains and their products |
| Yam (Dioscorea dumetorum) | Roots, tubers, plantains and their products |
| Yams (Dioscorea) | Roots, tubers, plantains and their products |
| Yergan seed | Pulses, seeds and nuts and their products |
| Yoghurt | Milk and milk products |
| Zobo drink | Indigenous dishes or beverages or beverages |

**Table S8: Food items per food group.**

| **Food group** | **Food items** |
| --- | --- |
| Beverages | Beverages; fermented beverages; green tea; kaffir beer; langbeen bossie (rooibos); tea; tea with fat cakes; wine |
| Cereals and their products | African rice (oryza glaberrima); barley; bran flakes; bread, white or dark; buns; carbohydrates, unrefined; cereal grains; cereals, wholegrain; corn flakes; couscous; fonio (digitaria exilis); grains; kaffir corn; local cereals; maize; maize (white and yellow); maize kernel; maize meal; maize products, fermented; mealie; mealie meal, stone ground; mealie rice; mealies (maize), green or dried; mealies, stamp; mealies, whole; millet; millet flour; millet, finger; oats; pearl millet; phaletshe or papa; porridge, maize; porridge, soft made with sorghum; porridges, starchy; rice; rice, white; sorghum; sorghum, sweet reed; teff (eragrotis tef (zucc) trotter; wheat |
| Composite dishes | Biscuits; fried foods; pasta; pastries; pizza; samosa; soups; stews |
| Eggs and their products | Eggs |
| Fats and oils | Fats; lard; margarine; oil palm crops; oils; olive oil; palm oil; vegetable oils |
| Fish, shellfish and their products | Crab; fish; fish, dried; seafood; shellfish |
| Foods for particular nutritional uses | Artemisia; fermented foods; starchy staples |
| Fruits and their products | Ackee; african breadfruit (treculia africana); african pear (dacryodes edulis); apple; bananas; bananas, sweet; bananas, yellow; berries; blackberry; buhunda; bushveld cherry (pappea capensis); buyeko; cactus pear; cape fig (ficus sur); cape holly (ilex mitis); carissa grandflora; chakata; dates; ensete (ensete vantricosum); figs; fruit; fruit juice; fruit, tropical; fruit, wild; guava; indigenous fruit; kaffir melon; koobo berry; lemons; lemons, unripe; lerotse; limes; mamonyi; mande; mangoes; marula (seasonal); matohwe; mazhanje; melon; melon seed (cucumeropsis mannii and citrullus lanatus); melon, wild; mfiru; ndobe; oranges; papaya; passion fruit; pawpaw; pineapples; plum, wild; prickly pear; sour plum; sungwa; tamarinds; tangerines; tomatoes; tree tomatoes; velvet tamarind (dialium guineense); watermelon; watermelon, wild; wild medlar (vangueria infausta) |
| Indigenous dishes or beverages | Bogobe; brukina; burukutu; deep-fried wheat flour dough; dolo; egusi; fufu; fura de nunu; gowé; isijabane; isijingi; isiphuphutho; kenkey; kunun-zaki; mahewu; nshima; ogi; sadza; soumbalà; tô; umnqutsho; wanderer's food; zobo drink |
| Insects, grubs and their products | Ants; caterpillars; edible insects including grasshoppers locusts crickets beetles caterpillars bees ants grasshoppers locusts crickets and termites; grasshoppers; insects; locusts; madora; mopane worms; spider whips; termites |
| Meat and meat products | African giant snail (archachatina marginata); animal foods; animal products; animals, wild; beef; beef, cooked (seswaa); beef, dried (biltong); birds; blood; camel; chicken; cow liver; goat; guinea fowl; lamb; liver; meat; meat, game from wildlife; meat, processed; meat, red; meat, salted; meat, traditional; meat, white; mice; mutton; offal; pigeon; pork; poultry; rabbit; serobe (offal); sheep; turkey |
| Milk and milk products | Dairy products; milk; milk (full cream); milk products; milk, cow; milk, fermented; milk, fresh; milk, goat; milk, raw cow; milk, sour; yoghurt |
| Pulses, seeds and nuts and their products | African locust bean (parkia biglobosa); african oil bean (pentaclethra tnacropltylla); african walnut (coula edulis); african yam bean; bambara groundnuts (vigna sub-terranea); beans; black bean; brown or white beans; cajanus cajan; caju; cashew; castor oil seeds; cluster beans; coconut; cowpea leaves (morogo wa dinawa); cowpeas (vigna unguiculata); dika; eland's wattle (elephantorrhiza elephantina); geocarpa groundnut (macrotyloma geocarpum); groundnut porridge; groundnuts (arachis hypogaea); hyacinth beans; kidney beans; kola; legumes; lentils; lima beans; macademia; mboco; mung beans; nÃ©rÃ© seeds; niÃ©bÃ©; nuts; oil seeds (rvingia gabonensis var excelsa); peas; pecan nuts; pigeon peas (cajanus cajan); pods; prosopis africana seeds; pulses; pumpkin bean; red bean (kunde); seeds; sesame; shea butter fruit; small green pea (thuu); small peas (thoroko or chirok); small round green pea (podzo); soy beans; soy milk; tree nuts; yergan seed |
| Roots, tubers, plantains and their products | African yam (dioscorea rotundata); banku; cassava (manioc); cocoyam; coleus potato; false yam; guinea yam; kaffir potato (coleus esculentus); local roots; native pototo; plantain; polony; potato, english; potato, native; potatoes; roots; starchy roots; sweet potato; tannia (xanthosoma sagittifolium); taro (colacasia esculenta); tiger nuts (yellow nutsedge); tubers; wood sorrel; yam (dioscorea dumetorum); yams (dioscorea) |
| Spices and condiments | African nutmeg; curcumin; ginger; spices |
| Sweets and sugars | Honey; sugar; sugar cane |
| Vegetables and their products | Abelmoschus esculentus; agaricus species; agushie (pumpkin seeds); amaranth; amaranth leaf; amaranthus thumbergii; auricularia auricular judae (bull) quÃ©l; avocado; baobab (adansonia digitata); baobab leaves; bean or pumpkin-leaf; beetroot; bitter gourd; bitter leaf (vernonia amygdalina); bitterberries; blackjack (bidens pilosa); bottle gourds; butternut squash; cabbage, cooked; cabbage, ethiopian (brassica carinata); cabbage, european; cabbage, fresh; carrot; cassava leaves; celosia argentea; chard; cleome gynandra; collybia butyracea (bull) p kumm; coprinus atramentarius (bull) fr; coprinus picaceus (bull) gray; corchorus olitorius; corchorus tridens; corchorus trilocularis; cordyceps sinensis; cucumber; cucumber, wild; cucumis hirsutus; daedaleopsis confragosa; derere (okra); desert mushroom; devil's thorn (sesamum eriocarpum); eggplant; fat hen (chenopodium album); fluted pumpkin; ganoderma lucidum; garden egg (african eggplant); garlic; gourd; green leafy vegetables; green vegetables; greens; grifola frondosa; gymnanthemum amygdalinum; herbs; herbs, wild; hericium erinaceus; hibiscus (hibiscus sabdariffa); horseradish tree leaves; indigenous plant foods; indigenous vegetables; jute mallow; kale; kenkiliba leaves; lactarius trivialis fr; lagneria vulgaris; laricifomes officinalis; lentinula edodes; lentinus squarrosulus mont; lenzites betulina; lettuce; macrolepiota species; moringa; mormodica balsamina; mushroom; nerÃ¨; nightshade, african (solanum species); nightshade, black (solanum nigrum); okra; olives; olives, wild; onion; oriolus versicolor; pepper; pepper (green/red/yellow); plant foods; plants, wild; pleurotus ostreatus; pleurotus pulmonarius; psathyrella atroumbonata pegler; pumpkin; purslane; rape (chomolia); rape greens; red sorrel; rooibos; roselle (hibiscus sabdariffa); rozites caperata; schizophyllum commune fr; sclerotium-forming pleurotus tuberregium; seaweed; senna leaves; soap aloe (aloe maculata); solanum macrocarpon; spiderplant (basella alba); spinach; squash; sumuka wiki; sweet potato leaves; tamarind tree leaves; termitomyces clypeatus heim; termitomyces globules; termitomyces globules heim and gooss; termitomyces mammiformis heim; termitomyces microcarpus (berk and br) heim; termitomyces robustus (beeli) heim; thistle, wild; tricholoma species; truffles; vegetables; vegetables, salad; vegetables, wild; volvariella esculenta; volvariella volvacea (bull) singer |

**Table S9. Amounts of food groups/food items.**

1. **Amounts reported as a percentage of total energy intake contributed to by food groups.**

| **Food Groups** | **Gibson et al., 2015** |
| --- | --- |
| **Fufu** | 16 |
| **Soups and stews** | 14 |
| **Kenkey** | 12 |
| **Banku** | 5 |
| **Plantain** | 5 |
| **White rice** | 4 |
| **Fish** | 4 |
| **White bread** | 4 |
| **Starchy porridges** | 4 |
| **Yam** | 4 |

1. **Amounts reported as average daily food group consumption (grams/day).**

| **Food Groups** | **Sodjinou et al., 2009*** |
| --- | --- |
| **Grains** | 787.1 (338.6) |
| **White bread and pasta** | 66.3 (62.9) |
| **Local roots and tubers** | 55.7 (63.1) |
| **Potatoes** | 0.4 (2.3) |
| **Legumes** | 69.8 (107.1) |
| **Nuts and seeds** | 3.3 (5.4) |
| **White meat** | 4.9 (9.7) |
| **Red meat** | 8.2 (14.0) |
| **Fish** | 66.6 (31.4) |
| **Eggs** | 3.1 (7.4) |
| **Milk** | 4.3 (8.5) |
| **Milk products** | 1.9 (6.9) |
| **Fruits** | 15.7 (34.5) |
| **Fruit juices** | 2.6 (7.1) |
| **Green leafy vegetables** | 110.4 (130.1) |
| **Other vegetables** | 189.6 (130.5) |
| **Oils** | 12.1 (7.7) |
| **Fats** | 5.6 (7.8) |
| **Sweets** | 16.6 (21.8) |
| **Soft drinks** | 23.3 (55.0) |
| **Fast food** | 0.0 (0.0) |

*Results expressed as mean (standard deviation)

1. **Amounts reported as average daily food group consumption (grams/day).**

| **Food Groups** | **Pretorius and Sliwa, 2011** |
| --- | --- |
| **Mabele (coarse)** | 40 |
| **Apple** | 160 |
| **Milk (full cream)** | 500 |
| **Sugar** | 15 |
| **Maize meal porridge** | 1000 |
| **Beans in tomato sauce** | 90 |
| **Chicken** | 90 |
| **Canola oil** | 10 |
| **Spinach** | 75 |
| **Carrots** | 75 |
| **Orange** | 180 |

1. **Amounts reported as servings/week consumed (servings/week).**

| **Food Groups** | **Frank et al., 2014*** | | | | |
| --- | --- | --- | --- | --- | --- |
| **Food group consumption by quintiles of weekly servings** | **T1** | **T2** | **T3** | **T4** | **T5** |
| **Juice** | 0.5 (0, 1.5) | 0.5 (0, 1.5) | 0.5 (0, 1.5) | 0.5 (0, 1.5) | 0.5 (0, 1.5) |
| **Sweets** | 0.5 (0, 0.5) | 0.5 (0, 0.5) | 0.5 (0, 0.5) | 0.5 (0, 0.5) | 0.5 (0, 0.5) |
| **Rice** | 3.5 (1.5, 7.0) | 3.5 (1.5, 6.3) | 3.5 (1.5, 7.0) | 3.5 (1.5, 7.5) | 5.5 (3.5, 7.0) |
| **Soft drinks** | 1.5 (0.5, 1.5) | 0.5 (0.5, 1.5) | 1.5 (0.5, 3.5) | 0.5 (0.5, 1.5) | 1.5 (0.5, 3.5) |
| **Vegetable oil** | 3.5 (1.5, 5.5) | 3.5 (1.5, 3.5) | 3.5 (1.5, 3.5) | 3.5 (1.5, 3.5) | 3.5 (1.5, 3.5) |
| **Milo** | 1.5 (0.5, 3.5) | 1.5 (0, 3.5) | 1.5 (0.5, 3.5) | 1.5 (0.5, 3.5) | 1.5 (0.5, 3.5) |
| **Red meat** | 1.5 (0.5, 3.5) | 1.5 (0.5, 3.5) | 1.5 (0.5, 3.5) | 1.5 (0.5, 3.5) | 1.5 (0.5, 3.5) |
| **Eggs** | 0.5 (0.5, 1.5) | 0.5 (0.5, 1.5) | 0.5 (0.5, 1.5) | 1.5 (0.5, 3.5) | 0.5 (0.5, 1.5) |
| **Margarine** | 0  (0,0.5) | 0  (0,0.5) | 0  (0,0.5) | 0  (0,0.5) | 0.5  (0,1.5) |
| **Fruits** | 1.5 (1.5, 3.5) | 1.5 (1.5, 3.5) | 3.5 (1.5, 3.5) | 3.5 (1.5, 3.5) | 3.5 (3.5, 3.5) |
| **Carrot** | 0.5 (0, 1.5) | 0.5 (0, 1.5) | 1.5 (0.5, 1.5) | 0.5 (0.3, 1.5) | 1.5 (0.5, 3.5) |
| **Lettuce** | 0.5 (0, 0.5) | 0.5 (0, 1.5) | 0.5 (0, 1.5) | 0.5 (0, 1.5) | 1.5 (0.5, 1.5) |
| **Milk** | 1.5  (0.5, 3.5) | 1  (0.5, 3.5) | 1.5  (0.5, 3.5) | 0.5  (0.5, 3.5) | 1.5  (0.5, 5.5) |
| **Poultry** | 0.5  (0.5, 1.5) | 0.5  (0.5, 1.5) | 0.5  (0.5, 1.5) | 0.5  (0.5, 1.5) | 0.5  (0.5, 1.5) |
| **Cucumber** | 0.5 (0, 0.5) | 0.5 (0, 0.5) | 0 (0, 0.5) | 0 (0, 0.5) | 0.5 (0, 1.5) |
| **Plantain** | 1.5  (0.5, 3.5) | 3.5  (1.5, 5.5) | 3.5  (1.5, 7) | 3.5  (3.5, 7.0) | 7  (3.5, 7.0) |
| **Green leaves** | 1.5  (0.5, 1.5) | 1.5  (1.5, 3.5) | 1.5  (0.5, 3.5) | 3.5  (1.5, 5.5) | 7  (3.5, 7.0) |
| **Beans** | 1.5  (0.5, 1.5) | 1.5  (0.5, 3.5) | 1.5  (0.5, 3.5) | 1.5  (0.5, 3.5) | 3.5  (1.5, 7.0) |
| **Garden egg** | 1.5  (1.5, 3.5) | 1.5  (1.5, 3.5) | 7  (1.5, 7.0) | 7  (7.0, 7.0) | 7  (7.0, 7.0) |
| **Fish** | 3.5  (3.5, 7.0) | 7  (4.5, 7.0) | 7  (7.0, 7.0) | 7  (7.0, 7.0) | 7  (7.0, 7.0) |
| **Maize (banku)** | 1.5  (1.5, 3.5) | 1.5  (1.5, 3.5) | 3.5  (1.5, 7.0) | 3.5  (1.5, 7.0) | 7  (7.0, 7.0) |
| **Palm oil** | 1.5  (1.5, 3.5) | 3.5  (1.5, 3.5) | 3.5  (1.5, 3.5) | 3.5  (3.5, 6.3) | 5.5  (3.5, 7.0) |
| **Okra** | 0.5  (0.5, 1.5) | 0.5  (0.5, 1.5) | 1.5  (0.5, 1.5) | 1.5  (0.5, 3.5) | 3.5  (1.5, 7.0) |

*Results expressed as median (IQR)

**Table S10. Characteristics of included reports (n=45).**

| **Author/Year** | **Country** | **Study design** | **Years/**  **period represented** | **Geographical location represented** | **Ethnicity** | **Method used to define diet** | **Definition of diet** |
| --- | --- | --- | --- | --- | --- | --- | --- |
| Afari-Sefa et al., 2015 | Tanzania | Qualitative study | NR | Eastern Africa | NR | NR | Traditional African diet |
| Aljefree and Ahmed, 2015 | Algeria, Egypt, Morocco, Libya, Tunisia | Systematic review | NR | Northern Africa | NR | NR | Traditional Northern African diet |
| Ashaolu and Adeyeye, 2021 | NR | Literature review | NR | Whole Africa | NR | Literature review | Traditional African diet |
| Auma et al., 2020 | Uganda | Cross-sectional study | 2017 | Eastern Africa | NR | Qualitative 24-hour dietary recall + hierarchical cluster analysis | Traditional African rural diet |
| Beagan and Chapman, 2012 | NA | Qualitative study | NA | NR | African Americans | NR | Traditional African diet |
| Casari et al., 2022 | Burkina Faso | Cross-sectional study | 2019 | Western Africa | African | FFQ + PCA | Traditional Burkinabe diet |
| Cisse et al., 2018 | Bamako, Mali | Observational study | NR | Western Africa, Southern Africa | Arican | NR | Traditional African diet |
| Claasen et al., 2015 | Republic of South Africa | Mixed-methods study | NR | Southern Africa | NR | NR | Traditional South African diet |
| Delisle, 2010 | Benin | Cross-sectional study | NR | Western Africa | NR | 2 or 3 non-consecutive 24-hour dietary recalls + cluster analysis | Traditional African diet |
| Dunne et al., 2022 | Nigeria | Literature review | NR | Western Africa | Native and migrant Ghanaians | NR | Traditional African diet |
| Fernandes et al., 2021 | NR | Literature review | NR | NR | NR | Literature review | Traditional African diet |
| Frank et al., 2014 | Ghana, Tanzania | Case-control study | 2007-2008 | Western Africa, Eastern Africa | Ghanaian | FFQ and 24-hour dietary recall + PCA | Traditional Ghana diet |
| Galbete et al., 2018 | Ghana | Cross-sectional study | 2010 | Western Africa | Ghanaian | Semi-quantitative food propensity questionnaire + PCA | Traditional Ghana diet |
| Gibson et al., 2015 | Ghana and UK | Observational study | NR | Western Africa | Native and migrant Ghanaians | 3-day food records + cluster analysis | Traditional African diet |
| Harris, et al., 2019 | Zambia | Literature review | NR | Southern Africa | NR | NR | Traditional African diet |
| Jannasch et al., 2017 | Ghana | Case-control study | 2007-2008 | Western Africa | NR | FFQ + PCA | Traditional Ghana diet |
| Katsidzira et al., 2018 | Zimbabwe | Case-control study | 2012-2015 | Southern Africa | NR | PCA | Traditional Zimbabwean diet |
| Kennedy and Reardon, 1994 | NR | Literature review | NR | Southern Africa | NR | NR | Traditional African diet |
| Kuhnlein and Johns, 2003 | Niger | Literature review | Early to mid 1900 | Western Africa, Southern Africa | NR | NR | Traditional West Africa diet; traditional Sahelian diet |
| Labadarios et al., 1996 | Republic of South Africa | Literature review | 1959-1994 | Southern Africa | Africans | Dietary survey | Traditional African diet |
| Legwegoh and Hovorka, 2016 | Gaborone, Botswana | Qualitative study | 2009-2010 | Southern Africa | NR | List of foods | Traditional diets in Botswana |
| Majova, 2014 | Republic of South Africa | Mixed-methods study | 2009 | Southern Africa | Nompumelelo community | Interviews, focus group and questionnaire | Traditional African diet |
| Makuse and Mbhenyane, 2011 | Republic of South Africa | Qualitative study | NR | Southern Africa | NR | List of foods via focus groups | Traditional African diet |
|  |  |  |  |  |  |  |  |
| Masarirambi et al., 2010 | Swaziland | Literature review | NR | Southern Africa | NR | NR | Traditional African diet |
| Masuku and Bhengu, 2021 | Republic of South Africa | Qualitative study | NR | Southern Africa | Emaphephetheni | Interviews | Traditional African diet |
| Oniang'o et al., 2003 | Whole Africa | Narrative review | NR | Whole Africa | African | Literature review | Traditional African diet |
| Osborn and Noriskin, 1937 | Nyasaland, Basutoland, Transkei, Bechuanaland, Elliotdale, Mqanduli, Chopi etc. | Literature review | NR | Southern Africa, Eastern Africa | Transkeian Bantu | Observed diet from the participants | Traditional African diet |
| Osuagwu, 2019 | NR | Literature review | NR | Western Africa | NR | NR | Traditional African diet |
| Pavan et al., 1999 | Tanzania | Cross-sectional study | 1997 | Eastern Africa | Bantu, Madilu | NR | Traditional Madilu diet |
| Pretorius and Sliwa, 2011 | Soweto and other African communities | The Heart Of Soweto (HoS) study | NR | Southern Africa | NR | NR | Traditional Black African diet |
| Rankoana, 2021 | Republic of South Africa | Qualitative study | NR | Southern Africa | NR | List of foods via interviews | Traditional African diet |
| Raschke et al., 2007 | Kenya, Tanzania, Uganda. | Cross-sectional study with a retrospective analysis component | 1930s-1960s | Eastern Africa | Luo, Kikuyu, Giriama, Samburu, Digo, Bahaya, Sukuma, Gogo, Hadza, Wasagara, Wakaguru, Wavidunda, Walu- guru, Nyamwezi, Iteso, Baganda, Acholi | 24-hour dietary recalls and weighed food records, retrospective analysis (cluster analysis ) of data collected in the original survey | Traditional Eastern African diet |
| Raschke and Cheema, 2008 | Kenya, Uganda, Tanzania | Literature review | 5000 years ago; 1000 years ago; colonial period | Eastern Africa | Ndorobo, Bantu and Nilotes | Literature review | Traditional Eastern African diet |
| Sodjinou et al., 2009 | Benin | Cross-sectional study | NR | Western Africa | NR | Three non-consecutive 24-hour food recalls, PCA | Traditional African diet |
| Spires et al., 2016 | Republic of South Africa | Literature review | NR | Southern Africa | NR | NR | Traditional African diet |
| Spires et al., 2021 | Republic of South Africa | Qualitative study | NR | Southern Africa | NR | NR | Traditional African diet |
| Steyn et al., 2012 | Kenya | Cross-sectional study | NR | Eastern Africa | NR | 24-hour dietary recall+ PCA | Traditional African diet |
| Tamang et al., 2020 | NR | Literature review | NR | Whole Africa | NR | NR | Traditional African diet |
| Vandebroek and Voeks, 2018 | NR | Literature review | NR | Southern Africa | NR | Literature review | Traditional African diet |
| Wagh et al., 2012 | Kenya, Tanzania | Cross-sectional study | NR | Eastern Africa | NR | NR | Traditional Maasai diet |
| Wiseman, 2015 | NR | Literature review | NR | Southern Africa | NR | NR | Traditional sub-Saharan African diet |
| Wrottesley et al., 2017 | Republic of South Africa | Cohort study | NR | Southern Africa | NR | PCA | Traditional African diet |
| Wrottesley et al., 2018 | Republic of South Africa | Cohort study | 2013-2016 | Southern Africa | Black South Africans | PCA | Traditional African diet |
| Zeba et al., 2014 | Burkina Faso | Cross-sectional study | 2010 | Western Africa | NR | A posteriori methods | Traditional African diet |
| Zotor et al., 2015 | Countries in Eastern Africa | Literature review | NR | Eastern Africa | NR | NR | Traditional East African diet |

FFQ, food frequency questionnaire; NR, Not reported; PCA, Principal component analysis

**Table S11. Quality assessment of included studies.**

| **Name (Year)** | **Description of what food items are included in the dietary pattern?** | **Description of what food groups are included in the dietary pattern?** | **Description of proportions, quantities, or frequencies of foods included in the pattern?** | **Clear description of the methodology used to identify the pattern?** | **Description of what geographical area(s) of the country are covered?** | **Description of sectors of the population represented?** | **Clear identification that the data/ descriptions are nationally or regionally representative** | **Description of what year(s) data were collected?** |
| --- | --- | --- | --- | --- | --- | --- | --- | --- |
| Afari-Sefa et al., 2015 | ⬤ | ⬤ | ⬤ | ⬤ | ⬤ | ⬤ | ⬤ | ⬤ |
| Aljefree and Ahmed, 2015 | ⬤ | ⬤ | ⬤ | ⬤ | ⬤ | ⬤ | ⬤ | ⬤ |
| Ashaolu and Adeyeye, 2021 | ⬤ | ⬤ | ⬤ | ⬤ | ⬤ | ⬤ | ⬤ | ⬤ |
| Auma et al., 2020 | ⬤ | ⬤ | ⬤ | ⬤ | ⬤ | ⬤ | ⬤ | ⬤ |
| Beagan and Chapman, 2012 | ⬤ | ⬤ | ⬤ | ⬤ | ⬤ | ⬤ | ⬤ | ⬤ |
| Casari et al., 2022 | ⬤ | ⬤ | ⬤ | ⬤ | ⬤ | ⬤ | ⬤ | ⬤ |
| Cisse et al., 2018 | ⬤ | ⬤ | ⬤ | ⬤ | ⬤ | ⬤ | ⬤ | ⬤ |
| Claasen et al., 2015 | ⬤ | ⬤ | ⬤ | ⬤ | ⬤ | ⬤ | ⬤ | ⬤ |
| Delisle, 2010 | ⬤ | ⬤ | ⬤ | ⬤ | ⬤ | ⬤ | ⬤ | ⬤ |
| Dunne et al., 2022 | ⬤ | ⬤ | ⬤ | ⬤ | ⬤ | ⬤ | ⬤ | ⬤ |
| Fernandes et al., 2021 | ⬤ | ⬤ | ⬤ | ⬤ | ⬤ | ⬤ | ⬤ | ⬤ |
| Frank et al., 2014 | ⬤ | ⬤ | ⬤ | ⬤ | ⬤ | ⬤ | ⬤ | ⬤ |
| Galbete et al., 2018 | ⬤ | ⬤ | ⬤ | ⬤ | ⬤ | ⬤ | ⬤ | ⬤ |
| Gibson et al., 2015 | ⬤ | ⬤ | ⬤ | ⬤ | ⬤ | ⬤ | ⬤ | ⬤ |
| Harris, et al., 2019 | ⬤ | ⬤ | ⬤ | ⬤ | ⬤ | ⬤ | ⬤ | ⬤ |
| Jannasch et al., 2017 | ⬤ | ⬤ | ⬤ | ⬤ | ⬤ | ⬤ | ⬤ | ⬤ |
| Katsidzira et al., 2018 | ⬤ | ⬤ | ⬤ | ⬤ | ⬤ | ⬤ | ⬤ | ⬤ |
| Kennedy and Reardon, 1994 | ⬤ | ⬤ | ⬤ | ⬤ | ⬤ | ⬤ | ⬤ | ⬤ |
| Kuhnlein and Johns, 2003 | ⬤ | ⬤ | ⬤ | ⬤ | ⬤ | ⬤ | ⬤ | ⬤ |
| Labadarios et al., 1996 | ⬤ | ⬤ | ⬤ | ⬤ | ⬤ | ⬤ | ⬤ | ⬤ |
| Legwegoh and Hovorka, 2016 | ⬤ | ⬤ | ⬤ | ⬤ | ⬤ | ⬤ | ⬤ | ⬤ |
| Majova, 2014 | ⬤ | ⬤ | ⬤ | ⬤ | ⬤ | ⬤ | ⬤ | ⬤ |
| Makuse and Mbhenyane, 2011 | ⬤ | ⬤ | ⬤ | ⬤ | ⬤ | ⬤ | ⬤ | ⬤ |
| Masarirambi et al., 2010 | ⬤ | ⬤ | ⬤ | ⬤ | ⬤ | ⬤ | ⬤ | ⬤ |
| Masuku and Bhengu, 2021 | ⬤ | ⬤ | ⬤ | ⬤ | ⬤ | ⬤ | ⬤ | ⬤ |
| Oniang'o et al., 2003 | ⬤ | ⬤ | ⬤ | ⬤ | ⬤ | ⬤ | ⬤ | ⬤ |
| Osborn and Noriskin, 1937 | ⬤ | ⬤ | ⬤ | ⬤ | ⬤ | ⬤ | ⬤ | ⬤ |
| Osuagwu, 2019 | ⬤ | ⬤ | ⬤ | ⬤ | ⬤ | ⬤ | ⬤ | ⬤ |
| Pavan et al., 1999 | ⬤ | ⬤ | ⬤ | ⬤ | ⬤ | ⬤ | ⬤ | ⬤ |
| Pretorius and Sliwa, 2011 | ⬤ | ⬤ | ⬤ | ⬤ | ⬤ | ⬤ | ⬤ | ⬤ |
| Raschke et al., 2007 | ⬤ | ⬤ | ⬤ | ⬤ | ⬤ | ⬤ | ⬤ | ⬤ |
| Raschke and Cheema, 2008 | ⬤ | ⬤ | ⬤ | ⬤ | ⬤ | ⬤ | ⬤ | ⬤ |
| Rankoana, 2021 | ⬤ | ⬤ | ⬤ | ⬤ | ⬤ | ⬤ | ⬤ | ⬤ |
| Sodjinou et al., 2009 | ⬤ | ⬤ | ⬤ | ⬤ | ⬤ | ⬤ | ⬤ | ⬤ |
| Spires et al., 2016 | ⬤ | ⬤ | ⬤ | ⬤ | ⬤ | ⬤ | ⬤ | ⬤ |
| Spires et al., 2021 | ⬤ | ⬤ | ⬤ | ⬤ | ⬤ | ⬤ | ⬤ | ⬤ |
| Steyn et al., 2012 | ⬤ | ⬤ | ⬤ | ⬤ | ⬤ | ⬤ | ⬤ | ⬤ |
| Tamang et al., 2020 | ⬤ | ⬤ | ⬤ | ⬤ | ⬤ | ⬤ | ⬤ | ⬤ |
| Vandebroek and Voeks, 2018 | ⬤ | ⬤ | ⬤ | ⬤ | ⬤ | ⬤ | ⬤ | ⬤ |
| Wagh et al., 2012 | ⬤ | ⬤ | ⬤ | ⬤ | ⬤ | ⬤ | ⬤ | ⬤ |
| Wiseman, 2015 | ⬤ | ⬤ | ⬤ | ⬤ | ⬤ | ⬤ | ⬤ | ⬤ |
| Wrottesley et al., 2017 | ⬤ | ⬤ | ⬤ | ⬤ | ⬤ | ⬤ | ⬤ | ⬤ |
| Wrottesley et al., 2018 | ⬤ | ⬤ | ⬤ | ⬤ | ⬤ | ⬤ | ⬤ | ⬤ |
| Zeba et al., 2014 | ⬤ | ⬤ | ⬤ | ⬤ | ⬤ | ⬤ | ⬤ | ⬤ |
| Zotor et al., 2015 | ⬤ | ⬤ | ⬤ | ⬤ | ⬤ | ⬤ | ⬤ | ⬤ |
